# Co-Expression of VAL- and TMT-Opsins Uncovers Ancient Photosensory Interneurons and Motorneurons in the Vertebrate Brain

**DOI:** 10.1371/journal.pbio.1001585

**Published:** 2013-06-11

**Authors:** Ruth M. Fischer, Bruno M. Fontinha, Stephan Kirchmaier, Julia Steger, Susanne Bloch, Daigo Inoue, Satchidananda Panda, Simon Rumpel, Kristin Tessmar-Raible

**Affiliations:** 1 Max F. Perutz Laboratories, University of Vienna, Vienna, Austria; 2 Research Platform “Marine Rhythms of Life,” University of Vienna, Vienna, Austria; 3 Research Institute of Molecular Pathology (I.M.P.), Vienna, Austria; 4 Centre for Organismal Studies, Heidelberg University, Heidelberg, Germany; 5 Regulatory Biology Laboratory, Salk Institute, La Jolla, California, United States of America; University of Geneva, Switzerland

## Abstract

The functional principle of the vertebrate brain is often paralleled to a computer: information collected by dedicated devices is processed and integrated by interneuron circuits and leads to output. However, inter- and motorneurons present in today's vertebrate brains are thought to derive from neurons that combined sensory, integration, and motor function. Consistently, sensory inter­motorneurons have been found in the simple nerve nets of cnidarians, animals at the base of the evolutionary lineage. We show that light-sensory motorneurons and light-sensory interneurons are also present in the brains of vertebrates, challenging the paradigm that information processing and output circuitry in the central brain is shielded from direct environmental influences. We investigated two groups of nonvisual photopigments, VAL- and TMT-Opsins, in zebrafish and medaka fish; two teleost species from distinct habitats separated by over 300 million years of evolution. TMT-Opsin subclasses are specifically expressed not only in hypothalamic and thalamic deep brain photoreceptors, but also in interneurons and motorneurons with no known photoreceptive function, such as the typeXIV interneurons of the fish optic tectum. We further show that TMT-Opsins and Encephalopsin render neuronal cells light-sensitive. TMT-Opsins preferentially respond to blue light relative to rhodopsin, with subclass-specific response kinetics. We discovered that *tmt-opsins* co-express with *val-opsins*, known green light receptors, in distinct inter- and motorneurons. Finally, we show by electrophysiological recordings on isolated adult tectal slices that interneurons in the position of typeXIV neurons respond to light. Our work supports “sensory-inter-motorneurons” as ancient units for brain evolution. It also reveals that vertebrate inter- and motorneurons are endowed with an evolutionarily ancient, complex light-sensory ability that could be used to detect changes in ambient light spectra, possibly providing the endogenous equivalent to an optogenetic machinery.

## Abstract 2

Evolutionarily conserved, nonvisual opsins appear to endow specific interneurons and motorneurons of the vertebrate brain with light sensitivity, suggesting that environmental light may be able to modulate information processing.

## Author Summary

The brains of vertebrates consist mainly of interneurons—neurons processing information coming from other neurons. Light information is believed to enter the brain through dedicated photoreceptor cells that are distinct from these processing cells, and motorneurons then relay the information to the musculature. Here we analyze two slowly evolving groups of vertebrate photopigment proteins, TMT-opsins and VAL-opsins, and find that both opsins are expressed in interneurons and motorneurons in medaka fish and zebrafish. Although these species diverged from a common ancestor over 300 million years ago and live in different habitats, the opsin localization is highly similar, suggesting a fundamental shared role for these proteins. Cultured cells expressing TMT-opsins respond to light, and electrophysiological recordings on adult brain slices identify a distinct set of light-sensitive interneurons. Based on our work, we argue that endogenous TMT- and VAL-opsin expression confers light-sensitivity on interneurons and motorneurons, and we propose two hypotheses. First, that modern vertebrate sensory neurons, interneurons, and motorneurons may derive from a common cell type that combined sensory, information processing and motor output functions. Second, that environmental light may modulate information transmission and processing in a distinct set of vertebrate interneurons and motorneurons.

## Introduction

Light impacts directly and indirectly on major processes of animal life, ranging from the entrainment of biological clocks 1,2 to the visual guidance of behavior [3]. It is commonly believed that such photosensory information enters into the vertebrate brain via dedicated sensory organs, is processed by interneurons, and can subsequently impact on motorneuron output.

However, nervous system evolution is thought to have started from few neuron types that combined sensory, integrative, and neurosecretory or motor output function within the same cells [4]–[7], similar to the “sensory-inter-motorneurons” of extant cnidarians, basally branching metazoans with a simple nerve net [8],[9]. This hypothesis predicts that the division between input, processing, and output is not a necessary requirement of higher brain centers, but a result of secondary evolution. Support for this notion stems from the occurrence of distinct cells that directly combine sensory functions (photo- or chemoreception) and neurosecretion. Such cells exist in both invertebrate and vertebrate brains [5],[10]. In vertebrates, photosensory-neurosecretory cells have been found in the pineal gland, the hypothalamus, and as interspersed cells in the lining of the brain ventricles in contact with the cerebrospinal fluid (CSF-contacting neurons) [5],[10].

Neurosecretion, however, is only used by a subset of brain neurons, and constitutes a slower mode of cellular communication. Currently, no evidence exists that vertebrate interneurons and motorneurons, constituting the majority of neuron types in the vertebrate brain, also possess comparable sensory modalities, even though environmental light penetrates deep into the brains of vertebrates [11], including mammals [12]. If “sensory-inter-motorneurons” were indeed present at the base of animal brain evolution, sensory-motorneurons and sensory-interneurons could still persist in the vertebrate central nervous system.

To address this hypothesis, we focused our attention on opsins, light-sensitive proteins that are present in animals ranging from cnidarians to humans. In recent years, a number of Opsins including Pinopsin, VAL-opsin, and Neuropsin (Opn5) have been detected in vertebrate brains [13]. These opsins have been shown to function as photopigments, and to be expressed in neurosecretory brain regions implicated in circadian or seasonal control [14]–[17]. A melanopsin expressed in the zebrafish larval preoptic area/hypothalamus has recently been suggested to mediate photokinesis, a light-seeking behavior triggered by loss of illumination [18].

In contrast to the aforementioned opsins, TMT-Opsin has been suggested to be broadly, potentially ubiquitously, expressed and to represent a light receptor mediating the entrainment of the peripheral circadian clock in zebrafish [19]. This presumptive function of TMT-Opsin and its specific confinement to teleosts is reflected in the name *teleost multiple tissue opsin* [20]. Whole mount in situ hybridizations on zebrafish larvae, however, localized *tmt-opsin* mRNA specifically to neurosecretory cells in the preoptic area/hypothalamus, challenging the hypothesis that TMT-Opsin would function as a peripheral light receptor [5].

We started our study by an investigation of TMT-Opsin, using the vertebrate model organisms zebrafish (*Danio rerio*) and medaka fish (*Oryzias latipes*). *In situ* hybridization experiments on adult brain sections reveal that *tmtopsins* are expressed not only in photoreceptive cells of the pineal, interneurons in the eye, and presumptive CSF-contacting neurosecretory cells. They are also expressed in brain nuclei neither connected to light sensation nor neurosecretion, such as the dorsal tegmental nucleus, the nucleus semicircular torus, the facial nerve nucleus, the periventricular layer of the tectum, and the granular layer of the olfactory bulb. In addition to the evolutionary conservation in expression over at least 300 million years, anti-TMTopsin1b staining reveals protein expression in neurons in these brain nuclei, suggesting that these opsins exhibit functions important for the animals. The neuronal nature of several *tmt-opsin*+ cells is confirmed by the co-expression of *cholineacetyltransferase* (*chat*), identifying them as interneurons and motorneurons. Anti-ChAT staining identifies typeXIV interneurons of Meek and Schellart in the teleost optic tectum among the *tmt-opsin+* cells. Expression of TMT-Opsins does not only render neuronal cells light sensitive in tissue culture, but we also show that interneurons at the position of typeXIV interneurons in isolated tectal slices of adult brains show electrophysiological responses to light.

Finally, we find that a subpopulation of *tmt-opsin*+ cells, including motorneurons of the facial nerve nucleus, co-express other *tmt-* and/or *val-opsins*, thereby endowing these supposedly non-sensory neurons with an unexpectedly complex ability to respond to light. The co-expression of ancient, functional inner brain opsins in specific inter- and motorneurons of the vertebrate brain lends strong support to the hypothesis that the diversity of vertebrate neurons evolved by multiplication and subfunctionalization of an ancient set of sensory-inter-motorneurons.

## Results

### TMT-Opsins and Encephalopsins Are Ancestral-Type Opsins Deeply Conserved across Vertebrates

**Figure 1:**
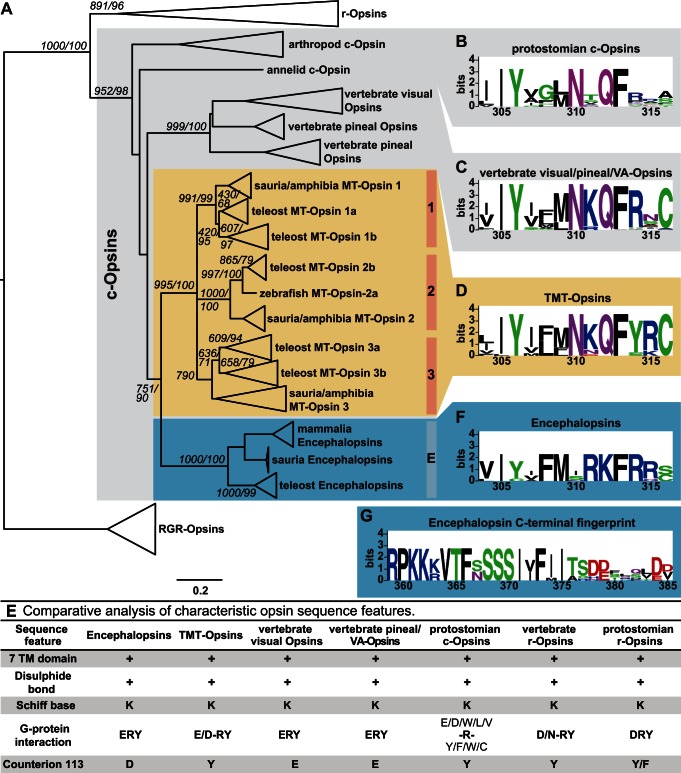
Phylogenetic and sequence analyses of TMT-Opsins and Encephalopsins. (A) Maximum likelihood (ML) and neighbor joining (NJ) trees group TMT-Opsins into three distinct subclasses conserved across vertebrates with high branch support. Encephalopsins form a fourth, closely related group. The topology of the NJ tree is shown, and support values are given as NJ/ML next to critical branches. Grey box, ciliary-type opsins; yellow, TMT-Opsins; blue, Encephalopsins. (B, C, D, F) Conserved sequence stretches in the c-terminus of the indicated opsin subfamilies. Numbers on *x*-axis refer to the amino acid positions in bovine rhodopsin (B, C, D, F) or human Encephalopsin (G). (E) Comparative analysis of characteristic opsin sequence features critical for photopigment function. Bovine rhodopsin is used as reference for counterion position. (G) Conserved sequence stretch in the c-terminus of Encephalopsins.

Two teleost TMT-Opsins (*tmtopsa*, *tmtopsb*) are currently reported in the literature [18],[20],[21]. Predicted protein sequence analysis of various vertebrate genomes revealed that vertebrates possess three distinct, conserved TMT-Opsin groups. In accordance with zebrafish nomenclature conventions, we termed these three groups TMT-Opsin 1, 2, and 3 and classified the already known TMT-Opsins into group 1, consequentially renaming them to *tmtops1a* and *tmtops1b*. Phylogenetic analysis revealed that TMT-Opsins and Encephalopsins are sister groups (Figure 1A). We jointly refer to both as the Encephalopsin-TMT-Opsin (ETO) family.

Closer sequence comparisons revealed that TMT-Opsins possess a conserved c-terminal domain typical for ciliary (c-)opsins ([22], Figure 1B–D), but share counterion residue with protostomian c-opsins and rhabdomeric (r-)opsins/melanopsins (Figure 1E). These features, together with their short branch lengths and overall strong sequence similarity with protostomian opsins, evidence that TMT-Opsins constitute an ancestral group of vertebrate c-opsins. In contrast, Encephalopsins differ in their counterion residue from TMT- and other opsins (Figure 1E). Furthermore, they are characterized by a different c-terminal fingerprint (Figure 1F) and possess an additional c-terminal domain, specifically conserved in Encephalopsins (Figure 1G), indicating that Encephalopsins might use different intracellular signaling pathways.

Genome and transcriptome analyses uncovered that the whole ETO family was already present at the base of vertebrates, as members of all four groups appear in teleosts, amphibians, and reptiles (Figure 1A). TMT-Opsin 2 orthologs are even more widely conserved: We identified a full-length TMT-Opsin 2 ortholog in the Opossum (*Monodelphis domestica*) genome (Figure S1), and partial sequences in the Platypus (*Ornithorhynchus anatinus*) and Wallaby (*Macropus eugenii*) genomes (Table S1). The fragmental nature of TMT-Opsins in platypus and wallaby likely reflects incomplete genome sequence assemblies, rather than mutated, partial TMT-Opsins. A screen of seven eutherian genomes revealed no TMT-Opsin orthologs (see Materials and Methods), suggesting that only Encephalopsins, but no other ETO members, are maintained in this mammalian subclass. This result is reminiscent of the loss of other opsin families in placental mammals, possibly linked to the lifestyle of their nocturnal ancestors [13].

### All ETO Groups Are Functional Photopigments Mediating Neuronal Photoreception

We next assessed if the encoded TMT-Opsins, as well as their sister group Encephalopsin, can indeed mediate photoreceptive functions.

All TMT-Opsin family members possess amino acid residues crucial for light detection (Figure 1E). We addressed their functional light sensitivity, relative absorption maximum, and response kinetics using a novel cell culture assay. Neuronal (Neuro-2A) and non-neuronal (HEK293) cells were transfected with representative *tmt-opsins*, and the cellular responses to light were recorded with an impedance-based real-time cell analyzer (RTCA). In this methodology cells grow on gold electrodes in a tissue culture plate, and a small alternating electrical current is repeatedly passed through the plate. The resistance to this current (impedance) depends on the surface area covered by the cells [23] and possibly their channel conductance. The impedance values were converted into a dimensionless parameter, the cell index (CI). CI changes have been used in various tissue culture assays to monitor G-protein signaling [24]–[27]. We reasoned that this assay could also be used to monitor light-dependent activation of opsins, given that opsins belong to the family of G-protein-coupled receptors (GPCRs) and that recent findings suggest that opsins lead to morphological changes of the photoreceptor membrane upon illumination [28].

**Figure 2:**
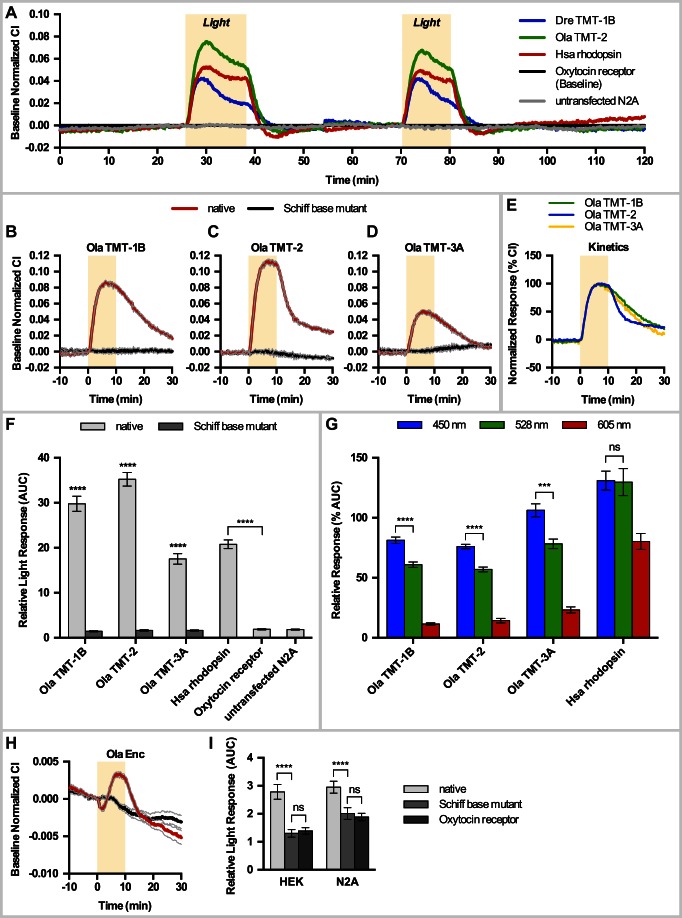
TMT-Opsins and Encephalopsin are functional light receptors. (A) Example traces of Neuro2A (N2A) cell stimulation by two consecutive light pulses of 12 min and 10 min. Data are presented as means (*N* = 4). (B–D) Traces of N2A cells transfected with medaka *tmt-opsins* (red) versus mutated (L294A) *tmt-opsins* (black), 10 min light stimulation. Data are presented as mean ± SEM (grey lines) (*N* = 4). (E) Different kinetics of TMT-2 (blue) compared to TMT-1B and TMT-3A in N2A cells (see also Figure S3). Baseline normalized CI values were normalized to the maximum and data are presented as means (*N* = 4). (F) Quantification of opsin-dependent N2A cell responses to light. Relative light responses are displayed as mean ± SEM (*N* = 72–136). (G) Quantification of opsin-dependent N2A cell responses to different spectra compared to human rhodopsin. Data represent mean ± SEM (*N* = 12–36). (H) HEK cells transfected with medaka *encephalopsin* (red) versus Schiff base mutant version (black). Data represent mean ± SEM (grey lines) (*N* = 32). (I) Quantification of Encephalopsin-dependent cell responses in HEK and N2A cells. Data represent mean ± SEM (*N* = 44–120); **** *p*<0.0001; *** *p*<0.0005; ns, not significant; yellow background box, light stimulation. See Figures S4 and S13 for analyses details.

We first established that our positive control, human rhodopsin responded reliably in this assay upon light exposure of the transfected cells (red trace, Figure 2A). The CI increased upon light exposure, reaching its maximum within 4 min and declining to baseline within a few minutes after cessation of light. Upon illumination with a subsequent pulse of light, the cells reproducibly exhibited a similar light response (Figure 2A). Although rhodopsin shows millisecond response in native photoreceptors, heterologous expression in mammalian cells typically produces a slower response [29], likely reflecting differences in photopigment density and associated proteins. The cells transfected with the GPCR human Oxytocin receptor or untransfected cells did not show any response to light (black and grey traces, Figure 2A). This established that the cell impedance assay can be used to detect light-dependent activation of opsins.

TMT-opsins localized to the cell membrane in transfected cells (Figures S2 and S9A,B). Like the cells transfected with rhodopsin, these cells showed a clear change of CI values during light exposure (Figure 2A). Individual TMT-Opsins exhibited characteristic responses distinguished by peak response and deactivation rate (Figure 2B–D). Opsins use *cis*-retinal bound to a Lysine residue (equivalent of K296 of bovine rhodopsin) as the light-sensitive chromophore and mutation of this residue abolishes light responses in rhodopsin [30]. Mutation of the orthologous Lysine residue to Alanine abolished the light response of all tested TMT-opsins (Figure 2B–D,F). This establishes that TMT-Opsins of all classes are functional photopigments likely forming a Schiff's base adduct with a *cis*-retinal.

Significant differences in response kinetics can be seen between the different TMT-Opsin representatives (Figure 2E). TMT-Opsins of group 2 showed a faster signal decay rate compared to opsins of group 1 and 3 (Figure 2E). Impedance response profiles have previously even been used to discriminate between different downstream pathways of GPCRs [25],[26]. As orthologous opsins from both fish species respond similarly in our assay (Figure S3), these differences indicate functional diversity among the TMT-Opsin groups in their signaling properties. In summary, our results reveal that all tested TMT-Opsins are functional photopigments (Figure 2F) and that response properties differ among groups. Consistent with our findings, zebrafish TMT-Opsin 1a has recently been shown to activate the enhancer of a zebrafish *period* gene in tissue culture in a light-dependent manner [19].

We next tested the relative spectral sensitivity of TMT-Opsins. We adjusted the light intensity using a set of band-pass and neutral density filters in order to activate human rhodopsin equally strong with a blue and a green filter, thus calibrating the setup to the rhodopsin absorption maximum of 497 nm ([31], Figure S4A–B). To minimize well-to-well variations, we compared CI changes to color light with a preceding white light stimulus (results of quantification given as relative response (%AUC), Figure S4C). All tested TMT-Opsins (medaka TMT-Opsin 1b, 2, and 3a) responded stronger to blue light (λ_max_ 450 nm), compared to green (λ_max_ 528 nm) or red (λ_max_ 605 nm) light, than human rhodopsin (Figures 2G and S4C–E). Also the absolute photon number values of the tested wavelengths were highly similar (see Figure S4 and Materials and Methods). None of the TMT-Opsins responded to near infrared light (950 nm, Figure S5).

We also tested the light sensitivity of Encephalopsin. The murine Encephalopsin ortholog has been shown to be specifically expressed in brain interneurons [32]. However, no evidence for a photoreceptor function of any Encephalopsin exists so far. With our assay we obtained clear light-dependent responses for medaka Encephalopsin in both Neuro-2A and HEK cells (Figure 2H,I). Even though the response maximum was lower than for TMT-Opsins, the response kinetics exhibited the typical trend seen for opsin activation (compare Figure 2H and Figure S6). Again, mutation of Lysine 296 to Alanine abolished the light-dependent response (Figure 2H,I), thus providing first evidence that Encephalopsins can function as light receptors.

### TMT-Opsins Show Specific Expression in Larval and Adult Medaka Fish Brain

In order to characterize the spatial distribution of the members of the ETO groups, we performed a systematic set of *in situ* hybridization (ISH) analyses. Medaka possesses two *tmt-opsins* from group 1 and 3, as well as one *tmt-opsin2* (Table S1). First, we examined expression patterns in medaka feeding larvae (stage 41/42), which revealed that all *tmt-opsins* are specifically expressed. We subsequently chose one member from each group for an in-depth characterization.

**Figure 3:**
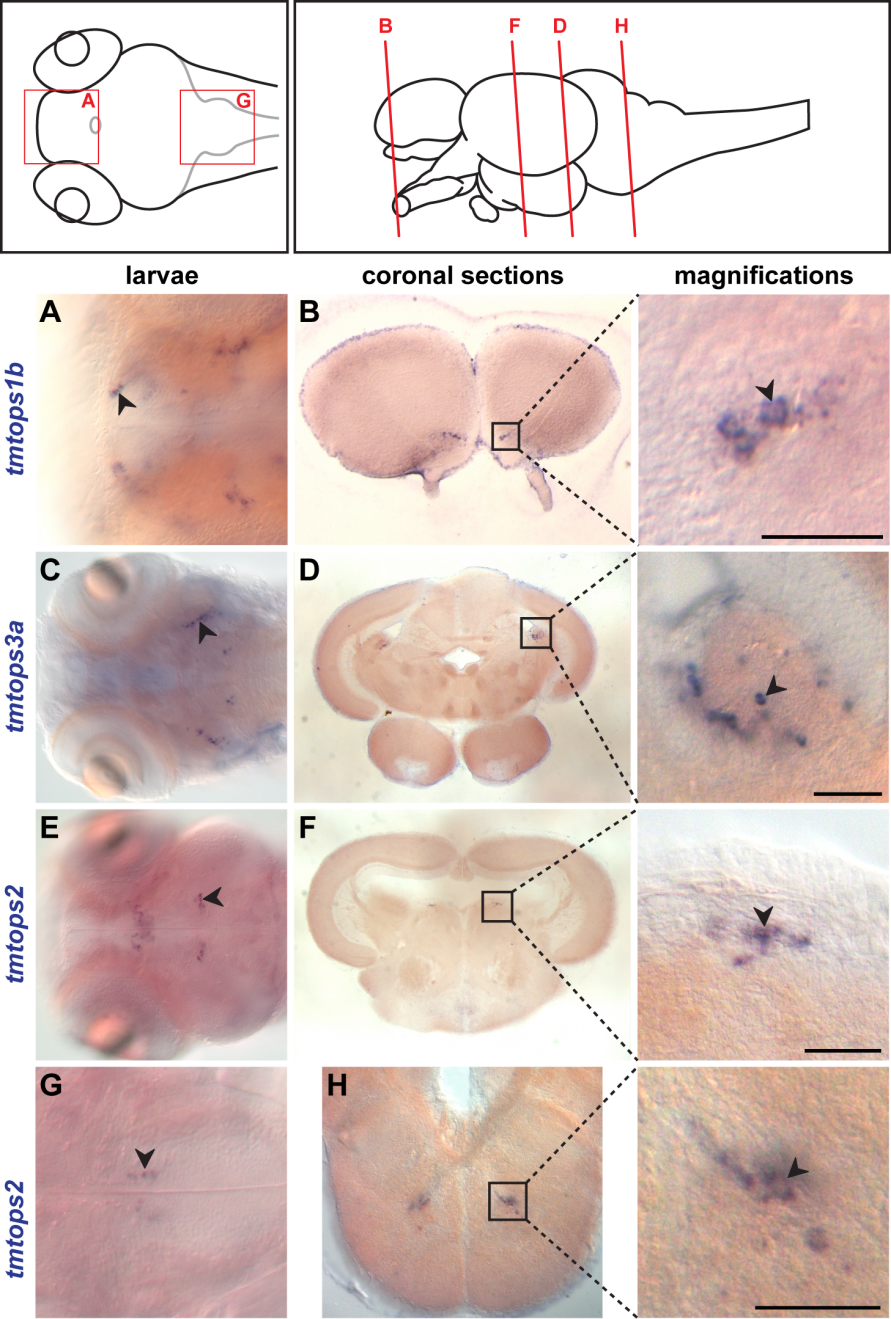
*TMT-Opsin* expression in inter- and motorneuron nuclei is maintained from larvae to adult stages. (Left topmost panel) Dorsal view of a schematized medaka larva; red boxes indicate positions of sections displayed below. (Right topmost panel) lateral view of an adult medaka brain, transversal planes corresponding to sections below. ISH on 7 dpf larvae (A, C, E, G) and coronal sections of the adult brain (B, D, F, H). Magnifications of boxed areas on the right; corresponding expression domains in larvae are indicated with arrowheads. Scale bars, 50 µm. Expression domains: *tmtops1b*, granular layer of the olfactory bulb (A, B); *tmtops3a*, semicircular torus (C, D); *tmtops2*, dorsal tegmental nucleus (E, F), facial nerve nucleus of the hindbrain (G, H).

All three analyzed *tmt-opsin* representatives exhibited highly specific expression domains in discrete brain nuclei, the eye and the pineal, but no detectable expression outside the nervous system (Figure S7A–D). The expression in the brain was not restricted to the diencephalon, the brain region typically associated with deep brain photoreceptors, but also included additional domains in the fore-, mid-, and the hindbrain (Figure S7A). In order to investigate if this specific expression is maintained in adulthood or rather represents a transient larval feature, we performed ISH on serial sections of the adult medaka brain. We find that the expression in the adult brain is as specific as in larvae (Figures 3 and S7E–G).

Systematic analysis of *tmt-opsin*+ cell clusters revealed four types of expression sites in the anterior nervous system. Two of these fit with current knowledge about photosensory brain cells:

On the one hand, we found *tmt-opsins* to be present in cells directly associated with light sensory organs: *tmtops1b* and *tmtops2* are expressed in amacrine cells (Figure S7B,C). Moreover, *tmtops2* is present in the iris and the annular ligament of the eye (Figure S7C), as well as in the pineal organ (Figure S7D,E).

On the other hand, several sites of *tmt-opsin* expression are also compatible with the notion that *tmt-opsins* are produced by inner brain sensory-neurosecretory cells. Previous analyses provided evidence for the existence of (at least in part CSF-contacting) photoreceptors in the vertebrate hypothalamus/preoptic area, as well as in the thalamus (summarized in [10]). Photoreceptors in these brain regions have been suggested to mediate photoperiodic reproductive physiology, as well as skin color regulation [10]. We detected *tmtops2* in the preoptic area (Figure S7E–F) and *tmtops1b* and *2* close to the third ventricle in the anterior and ventromedial thalamic nucleus (Figure S7E–G). Their position in close proximity to the cavity of the third ventricle is highly reminiscent of the position of *val-opsin+* neurons in zebrafish that contact the central spinal fluid [10] [33].

### TMT-Opsins Are Expressed in Inter- and Motorneurons on mRNA and Protein Level

Whereas the aforementioned expression sites concern domains and cell types previously associated with light sensation, we also identified two additional, unexpected categories of opsin-expressing cells that have so far not been associated with light-dependent functions.

**Figure 4:**
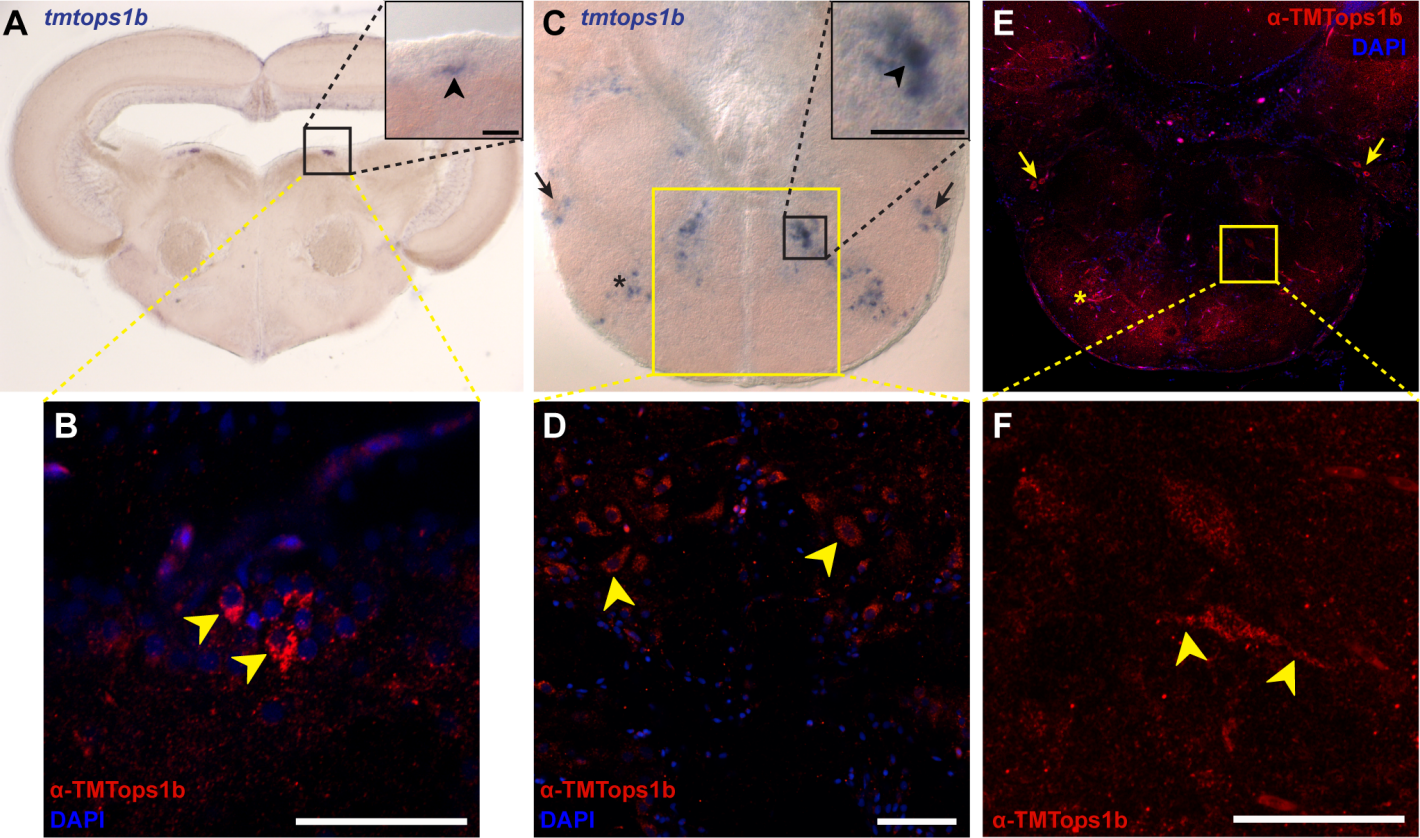
*TMT-Opsin 1B* is expressed in inter- and motorneuron nuclei on mRNA and protein level. ISH (A, C) and immunohistochemistry (B, D, E, F) of TMT-Opsin 1b on coronal adult medaka brain sections. Magnification of black boxes in insets. Scale bars, 50 µm. (A) mRNA expression of *tmtops1b* in the dorsal tegmental nucleus. (B) Protein expression of TMTopsin1b in cells of the dorsal tegmental nucleus, the same area as in (A). Arrowheads indicate TMTopsin1b+ cells. (C) Multiple domains of mRNA expression of *tmtops1b* in the hindbrain. (D) TMTopsin1b+ cells localize to sites of mRNA expression indicated by a yellow box in (C). (E) Overview of TMTopsin1b protein expression in the hindbrain. Arrows and asterisks indicate protein expression domains that correspond to mRNA expression in (C). (F) Magnification of box in (E), *z*-stack: 13.87 µm. Note the projections (arrowheads) extending from a TMTopsin1b+ cell verifying its neuronal nature.

First, we identified *tmt-opsins* in specific interneuron nuclei: We detected *tmtops1b* in the inner cellular layer of the olfactory bulb (Figure 3A,B) and *tmtops3a* in the second layer of the semicircular torus (Figure 3C,D). The semicircular torus receives input from lateral line and auditory fibers in vertebrates [34]. In addition, *tmtops2* and *1b* are expressed in the dorsal tegmental nucleus of the midbrain (Figures 3E,F and 4A), a structure receiving telencephalic input in fishes [34]. *Tmtops1b* is also present in multiple hindbrain cell clusters, part of which we identified to belong to the reticular formation (Figure 4C).

Next, *tmt-opsins* also label at least one motorneuron nucleus: *tmtops2 and 1b* positive cells are present in the nucleus of the facial nerve located in the hindbrain (Figures 3G,H and 4C). This nucleus innervates the vertebrate facial musculature [34].

Comparison of expression domains between feeding medaka larvae (arrowheads in Figure 3A,C,E,G) and adults (boxes in Figure 3B,D,F,H) revealed that most domains present in larvae can also be identified in the adult brain, suggesting stable expression of TMT-Opsins throughout the animal's life. Sense controls did not show any staining in the brain nuclei mentioned above (Figure S8). Using an antibody raised against TMT-Opsin 1b, we confirmed that opsin protein is present in cells of the identified inter- and motorneuron nuclei (Figure 4, see Figure S9 for controls, including pre-immune serum and peptide-neutralized antibody controls).

**Figure 5:**
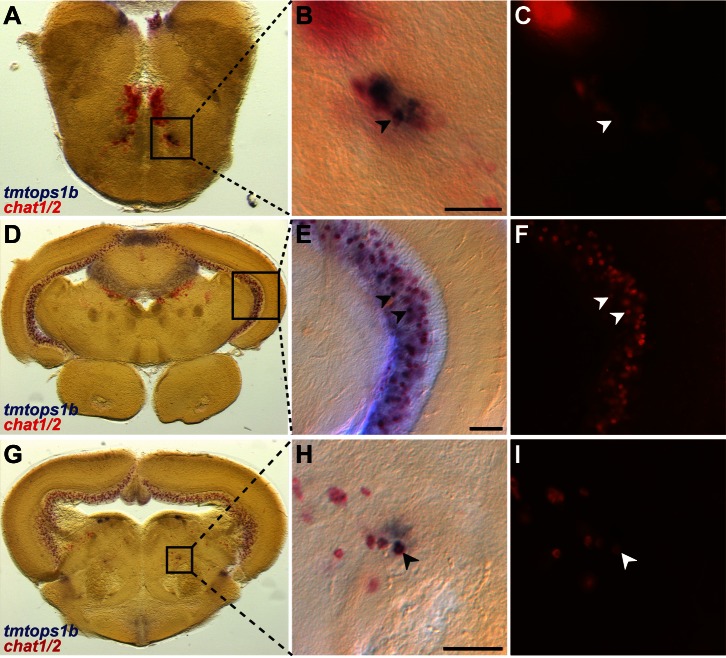
Co-expression of *tmt-opsins* with *choline acetyltransferase* in distinct inter- and motorneurons. Two-color ISH of *tmtops1b* (blue) and *chat1/2* (red/red fluorescence) on coronal adult medaka brain sections. Transversal planes of (A), (D), and (G) correspond to planes in Figure 3H, D, and F, respectively. (B, E, H) Magnification of boxed areas. (C, F, I) Fluorescent images of *chat1*/*2* staining. Arrowheads, co-expressing cells. Scale bars, 50 µm. Co-staining in facial nerve motorneurons (A–C), in interneurons of the periventricular grey zone of the tectum (D–F), and in interneurons of the rostral tegmental nucleus (G–I). Note that fluorescent signal of *chat1/2* expression can be quenched in areas of strong *tmt-opsin* staining (for higher magnification see Figure S10).

We next examined the neuronal identity of these opsins-expressing cells. Especially in the hindbrain, TMTopsin1b localizes to the beginning of projections (Figure 4F), strongly suggesting that TMTopsin1b+ cells are neuronal. Furthermore, we used *chat1* and *chat2*, both orthologs of the acetylcholine synthesizing enzyme, as markers specifically demarcating several interneuron populations in the teleost fore- and midbrain, as well as motorneurons in the facial nerve nucleus in the hindbrain [35]. We found *chat1/2* co-expressed with *tmtops1b* and *tmtops2* in the facial nerve nucleus, suggesting a motorneuron nature of *tmt-opsin+* cells (Figures 5A–C and S10A–C). Co-staining supports that *tmt-opsins* are present in interneurons of the tectum (XIV-type neurons of Meek and Schellart [35]; Figures 5D–F and S10D–F), as well as in the rostral tegmental nucleus (Figure 5G–I).

### A Subset of Tectal Interneurons at the Position of *TMT-opsin+* Neurons Is Light Sensitive

**Figure 6:**
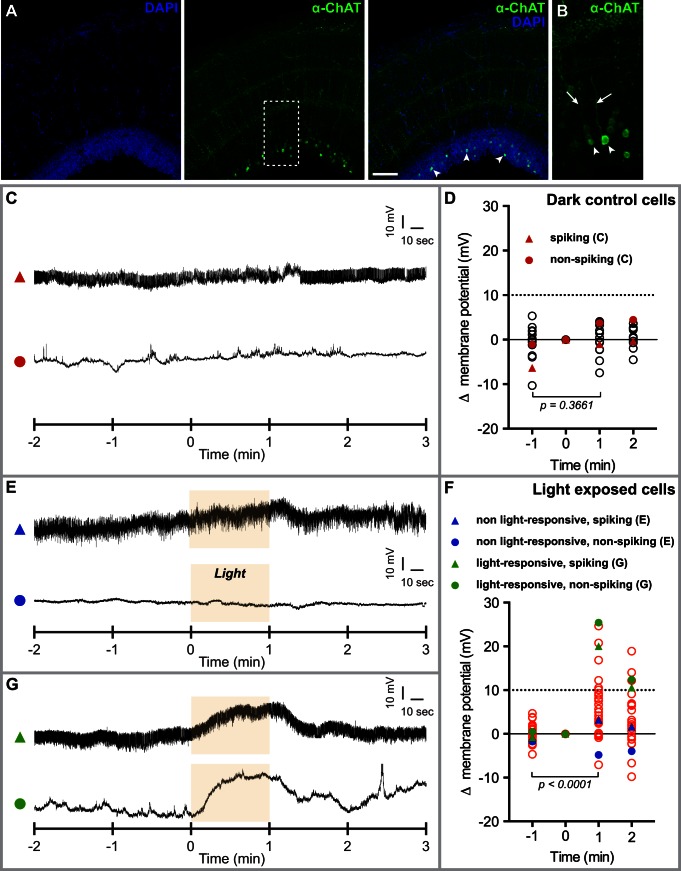
A subset of tectal interneurons are intrinsically light sensitive. (A) Confocal ISH images of anti-ChAT staining (green) in the tectum of coronal whole-brain slices. Z-stack, 25.55 µm (B) Magnification of boxed area in (A). Z-stack, 25.55 µm; arrowheads, ChAT-positive interneurons; arrows, neurites projecting from ChAT-expressing interneurons. (C) Representative membrane potential traces from two tectal interneurons recorded under total darkness. (D) Membrane potential changes of interneurons recorded under darkness (*N* = 14). Values were calculated in relation to the reference point (0 min). No significant difference can be observed between t = −1 and t = +1. Example traces in (C) of one spiking (triangle) and one nonspiking (circle) neuron highlighted in red. Note that delta membrane changes of cells recorded under darkness never get close to 10 mV (dashed line). (E) Representative membrane potential traces from single non-light-responsive interneurons exposed to light (yellow box). (F) Membrane potential changes of interneurons exposed to 1 min light (*N* = 28). Example traces in (E) of one intrinsically spiking (triangle) and a nonspiking (circle) neuron that do not respond to light are highlighted in blue. Light-responsive (circle) and intrinsically spiking light-responsive (triangle) interneurons in (G) are marked in green. A significant difference can be observed between t = −1 and t = +1. (G) Representative membrane potential traces from single light-responsive interneurons exposed to light. The *p* values were assessed by paired Student's *t* test.

Given that TMT-Opsins can render neuronal cells light-sensitive in tissue culture, their specific expression in inter- and motorneurons suggested that there might be intrinsically light-sensitive interneurons present in the medaka fish. To test this hypothesis, we turned to the optic tectum, as our expression analyses indicated that this structure harbors *tmtopsin*-positive cells in a relatively defined morphological region (see above). Antibody staining against ChAT confirmed the interneuronal nature of the *tmtopsin1b* and *tmtopsin2-*expressing cells, as clear neurite projections were visible (Figure 6A,B). The morphology of teleost tectal interneurons is unipolar, a morphology otherwise typical for insect neurons [36]. Our analysis also revealed that the ChAT+ cell bodies are typically located close to the ventricle (Figure 6A,B).

Next, we established patch-clamp whole-cell recordings from the adult tectum. To exclude any possible input from known photoreceptors in the retina or the pineal, we recorded from tectal slices (Figure S11). In the absence of any morphological feature indicative of light-sensitive neurons, we randomly recorded from neurons, expecting only a small fraction of the recorded units would show photosensitivity. We first recorded neurons (at the soma) under darkness. Their response was quantified by calculating the membrane potential change relative to the reference point just before the cells would be illuminated in the light response test experiment (time = 0). In darkness, changes in membrane potential remained well below 10 mV and showed no significant difference between the analyzed time points (two representative example traces in Figure 6C, whole dataset in Figure 6D, *N* = 14). Next, we tested light-sensitivity by administering a 1 min light pulse after 2 min of recording under darkness. The majority of neurons (*N* = 22) showed no change in membrane potential in response to light beyond what had been observed for the dark control (two representative example traces in Figure 6E, whole dataset in Figure 6F), thus excluding the possibility that illumination causes any systemic bias in our measurements. However, we identified six neurons whose membrane potential remained stable during darkness, but changed strongly upon illumination (two representative example traces in Figure 6G, whole dataset in Figure 6F). Their membrane potential during darkness was comparable to the average membrane potential of the light-insensitive neurons (average, −30.91 mV (+/−2.3 mV); green triangle neuron, −30.12 mV; green circle neuron, −45.59 mV), but increased strongly upon illumination (Figure 6F). This led to a significant difference in the membrane potential before and during illumination for the recorded set of light exposed interneurons (Figure 6F). These data strongly suggest that a small fraction of tectal interneurons responds to light, consistent with the observed expression of TMT-Opsins.

Our finding of light-responsive interneurons is consistent with previous observations. A study on the optical control of zebrafish behavior by optogenetics reported that 14% of the recorded neurons in wild-type larval brain responded endogenously to illumination [36]. Whereas these fish lacked functional eyes, the pineal organ was still present. Although the recorded neurons were located outside of the known projection area of pineal neurons, the possibility remained that the recorded light response could have been conveyed indirectly by the pineal [36]. In the case of the tectal slices, this possibility can be fully ruled out. Even if the recorded response were a circuit response, the only neuron types present in the tectal slice are interneurons.

### TMT- and VAL-Opsin Co-expression Suggests Complex Photosensory Abilities of Vertebrate Inner Brain Nuclei

**Figure 7:**
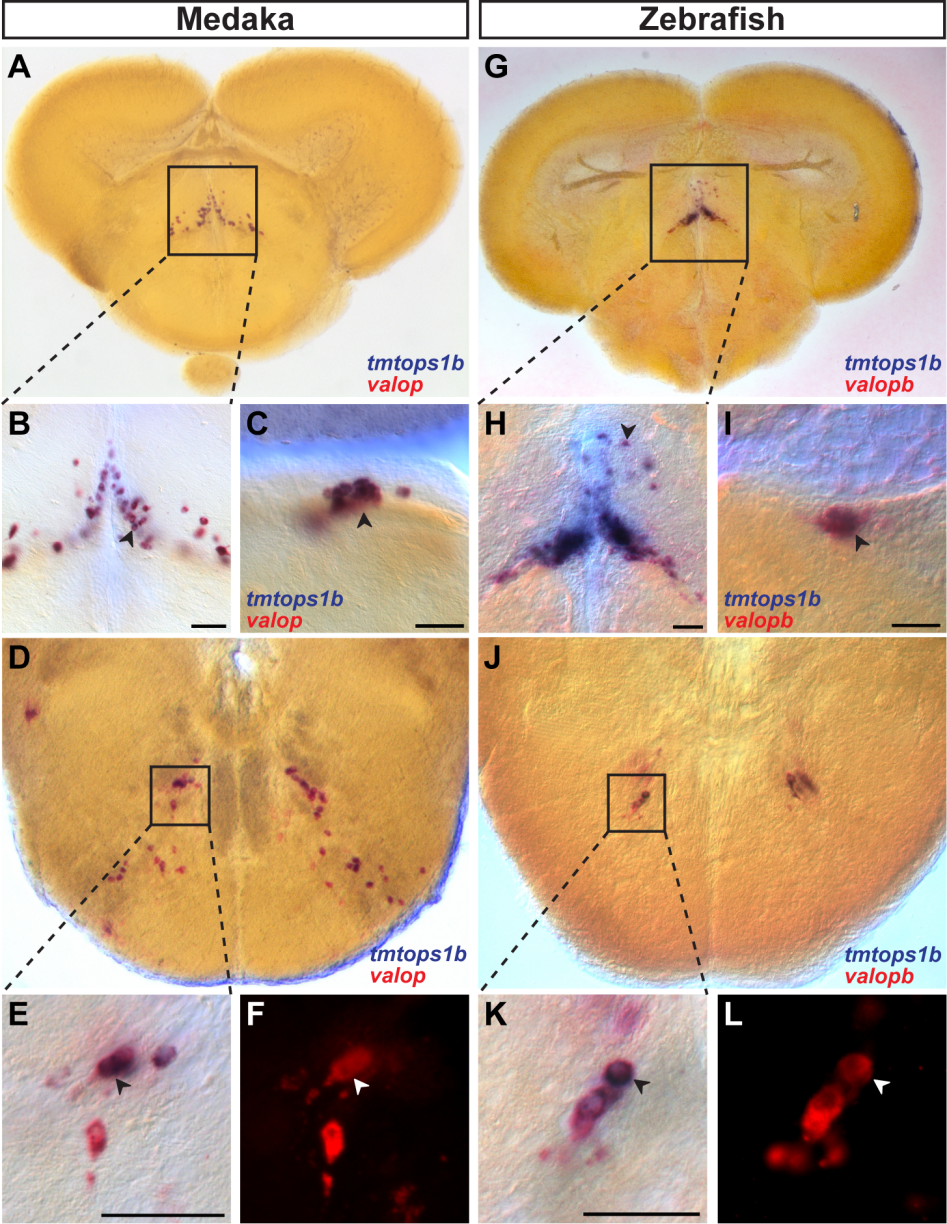
*Tmt*- and *val-opsin* co-expression domains in inter- and motorneuron cluster are conserved in vertebrates. Two-color ISH of *tmt-opsin1b* (blue) and *val-opsins* (red/red fluorescence) on coronal medaka (A–F) and zebrafish (G–L) sections. Magnifications are indicated as boxes. Scale bars, 50 µm. *Tmtops1b* and *valopb* co-expression in the central posterior thalamic nucleus of medaka (A–B) and zebrafish (G–H), in the dorsal tegmental nucleus in medaka (C) and zebrafish (I) and in the facial nerve nucleus in medaka (D–F) and zebrafish (J–L).

One well-known example of inner brain ciliary opsins are VA/VAL-Opsins shown to express in neurons close to the third ventricle in the thalamus and hypothalamus of fishes [10] and birds [15]. Searching for putative VAL-Opsin orthologs in medaka fish, we found one VAL-Opsin, most closely related to VAL-Opsin A. In order to investigate if *tmt-* and *val-opsin* expression demarcates common or distinct vertebrate brain nuclei, we performed two-color in situ hybridizations on serial sections of the medaka adult brain. In most cases we detected a strong correlation of *val*- and *tmt-opsin* expression patterns (Figure 7A–F). This was most obvious when we compared *valop* and *tmtops1b* (Figure 7A–F). We found such co-expression in presumptive CSF-contacting neurons in the central posterior thalamic nucleus (Figure 7A–B), but also in the dorsal tegmental nucleus (Figure 7C), as well as in motorneurons of the facial nerve nucleus in the hindbrain (Figure 7D–F).

In summary, our expression data provide strong evidence that several specific neuronal clusters in the inner vertebrate brain, including inter- and motorneurons, express a combination of multiple *opsin* genes. Given the different spectral preferences of co-expressed TMT- and VAL-Opsins, this co-expression suggests that such cells are endowed with a remarkably complex ability to respond to light.

### TMT- and VAL-Opsin Brain Expression Domains Are Conserved Across Teleosts

In order to investigate if the specific expression is a general, species-independent feature of *tmt-opsins* and *val-opsins*, we extended our analysis to their orthologs in zebrafish. Medaka and zebrafish diverged about 300 million years ago [37], a similar evolutionary distance as between mammals and birds (≈310 million years, [38]). In addition to their evolutionary divergence, both teleost species originate from different habitats and display different life styles. Japanese medaka fish encounter strong seasonal (winter/summer) differences and exhibit photoperiod-dependent reproduction cycles [39],[40], whereas subtropical zebrafish use temperature and food availability rather than photoperiod to discriminate between monsoon versus nonmonsoon seasons [41],[42].

Zebrafish possesses two paralogs of each TMT-Opsin group and one Encephalopsin. Consequently, two zebrafish TMT-Opsin 2 orthologs, TMT-Opsin 2a and TMT-Opsin 2b, exist (Table S1). Like medaka *tmtops2*, *Dre tmtops2a* is expressed in the amacrine layer and the annular ligament of the eye (Figure S12A,B), the pineal (Figure S12B,C), as well as in the facial nerve nucleus of the hindbrain (Figure S12D). Several zebrafish *tmt-opsins* are expressed in the dorsal tegmental nucleus of the midbrain, as is the case in medaka (Figure S12E–G, compare to Figure 3E,F,4A). Similarly, zebrafish *tmtops1b* was detected in the hindbrain (Figure S12H) (nucleus of the facial nerve) and thalamus (central posterior thalamic nucleus) (Figure S12I), just like its counterpart in medaka.

In addition to *tmtopsins*, zebrafish possess two *val-opsin* paralogs (A and B) expressed in several domains in the larval zebrafish brain and eye [43], as well as in the central posterior thalamic nucleus of the adult zebrafish brain [33]. Performing comparative expression analyses between *tmt-* and *val-opsins*, we find that also in zebrafish the majority of *tmt-opsin* expressing cells expressed *val-opsins* (Figure 7G–L) in a conserved patterned compared to medaka (Figure 7A–F).

Overall, our data argue that the co-expression of *tmt-* and *val-opsins* and the majority of expression sites are conserved across about 300 million years of evolution. This suggests that both opsin families have a critical function in the expressing cells, and that the conservation of *tmt-* and *val-opsins* expression domains might well extend to other vertebrates.

## Discussion

### Intrinsically Photosensitive Motor- and Interneurons (IPMIN)?

Over the past 10 years, expression of an increasing number of opsins (typically with ancestral sequence features) has been detected in vertebrate brains. Whereas the prevailing paradigm is that such inner brain opsins are involved in seasonal regulation, some of the investigated species such as zebrafish display little or no overt photoperiodicity [41],[42]. This is compatible with the idea that inner brain opsins also serve other, more fundamental functions in the vertebrate brain.

Our study provides strong evidence that in addition to neurosecretory cells, specific subsets of inter- and motorneurons are light-sensitive. As we show, not only the sequence of TMT-Opsins, but also their expression sites are highly conserved across evolution, arguing that these genes are involved in critical functions of the vertebrate brain. The fact that each of the tested inner brain opsins is able to render cultured neuronal cells photoreceptive, and that response kinetics differ between subgroups, suggests that light can influence distinct cellular properties of inner brain opsin-expressing neurons. Consistent with this hypothesis, we show that some neurons at the position of *tmt-opsin* expressing cells in the adult fish tectum directly respond to light by changes in membrane potential. This puts forward the possibility that information processing in some vertebrate inter- (and motor-)neurons can be influenced directly by environmental light. This hypothesis is further backed by our finding that TMT-Opsins differ from VAL-Opsins [33] in spectral preference (blue versus green light, respectively), allowing these neurons to perceive light of wider spectral range. To our knowledge, co-expression of opsins with different kinetics and spectral sensitivity has so far only been observed for neurons in the retina and pineal/parietal eye. In these organs, co-expression is suggested to mediate color vision (eye photoreceptors, e.g. [44]), adaptation to background irradiation (eye horizontal cells, [45]), and to improve dusk/dawn detection (parietal eye) [46]. Based on these examples, the co-expression of inner brain opsins in inter- or motorneurons might represent a fast possibility to adapt behavior to unpredictable environmental light changes. Given the specificity of *opsin*-expressing neurons, light-dependent responses are likely to be fast and highly cell-specific, in similarity to the concept of “optogenetic” switches, but in contrast to typical hormonal responses influencing tissues within the entire body.

### Sensory-Interneurons and Sensory-Motorneurons: “Living Fossil Neurons” of the Vertebrate Brain?

Our data not only support the notion that inner brain opsins have conserved functions in vertebrate brains, but are also compatible with the hypothesis that higher order brain centers initially evolved from multifunctional “sensory-inter-motorneurons,” as they can be found in extant cnidarians [8],[9]. Multifunctional neurons might therefore not just represent “add-ons” to existing brain circuits, but even relate to their ancient functions. This is compatible with the observation that both TMT-Opsins (this study) and VAL-Opsins [47],[48] display most ancient-type sequence features when compared with other opsins, and that the superfamily of c-opsins can already be distinguished in cnidarians [49]. This view also has implications for the interpretation of the few known light-dependent modulatory functions, such as the direct influence of light on the electrophysiological response of retinal interneurons [45]. This effect—mediated by VAL-Opsins and/or melanopsins—was suggested to be a functional specialty of the retina to integrate overall irradiance in the process of light perception [45]. In addition, it has been thought to reflect the evolutionary relationships of retinal interneurons to ciliary-type versus rhabdomeric-type photoreceptors [50]. Our data suggest that both the light sensory ability of interneurons, as well as their evolutionary relationship to ciliary photoreceptors might also hold true for interneurons of the inner brain and are no specialty of the eye.

## Materials and Methods

### Zebrafish and Medaka Fish Husbandry

All animal work was conducted according to Austrian and European guidelines for animal research. Zebrafish (*Danio rerio*) strains AB and Mitf-a and medaka fish (*Oryzias latipes*) strains CAB and Heino were kept in a constant recirculating system at 28°C on a 16 h light/8 h dark cycle. Collected embryos were kept at 28°C until hatched.

### Cloning of *tmtops* cDNA Sequences

A BLAST search was performed with the Zebrafish *tmtops1a* gene sequence against medaka and zebrafish genomes. Full-length coding sequences were predicted with FGENESH+ (softberry.com) using *Takifugu rubripes* TMT-Opsin as input protein sequence. New candidates were amplified via nested RT-PCR and 3′ and 5′ RACE (Clontech) from medaka and zebrafish cDNA libraries and sequence verified. Total RNA was extracted from larvae of mixed stages using the RNeasy kit (Qiagen), and reverse transcription was performed using the Transcriptor High Fidelity cDNA Synthesis Kit (Qiagen).

### Phylogenetic Analysis

Sequences were aligned using the MAFFT alignment algorithm (http://www.ebi.ac.uk/Tools/mafft/index.html). The resulting alignments were subsequently used to generate Neighbor Joining (NJ) and Maximum Likelihood (ML) trees. NJ trees were generated using ClustalX software [51], correcting for multiple substitutions and bootstrapped by 1,000 repetitions. For ML analysis, consensus trees were calculated (using TREE-PUZZLE [52] (v5.3)) from 100 trees generated with IQPNNI [53] (v3.3.2) using the BLOSUM62 substitution matrix and four categories of gamma-distributed substitution rates. Accession numbers of protein sequences used: Q17053, P28682, P60015, P51476, P06002, P08099, P04950, P08255, P91657, O01668, P22328, P28683, Q25157, Q25158, Q9UHM6, P08100, P22671, P35361, P24603, O02464, Q9WUK7, Q9QXZ9, P09241, O15973, Q98980, O42490, P51475, Q94741, O16005, O13018, P31356, P29403, O57422, P35356, XP_003228056.1, XP_003218813.1, AAD32622.1, XP_312503.4, XP_312502.2, NP_001035057.1, NP_786969.1, AF233520_1, AAD38035.1, EHJ69785.1, CBN82169.1, BAA94289.1, AAR14681.1, AAR14684.1, AAR14682.1, AAR14682.1, AF140242_1, NP_067315.1, XP_003439339.1, XP_003453327.1, AF077189_1, AAV63834.1, CAC86665.1, NP_001017877.1, NP_001026387.1, NP_001012738.1, NP_001178862.1, NP_001029021.1, EFA01685.1, CAG09827.1, NP_001165363.1, XP_002932669.1, XP_002933418.1, XP_001952294.2, XP_001234389.1, XP_426139.2, XP_003204046.1, XP_003218388.1, XP_003218813.1, and XP_003410963.1. Predicted opsin sequences are given as Ensemble coordinates in Table S1.

The following eutherian genomes were screened for TMT-Opsin orthologs as described above: *Tursiops truncatus*, *Bos taurus*, *Loxodonta africana*, *Mus musculus*, *Rattus norvegicus*, *Pan troglodytes*, and *Homo sapiens*.

### Real-Time Cell Analysis Assay

Neuro-2a (N2A) and HEK293 (HEK) cells (American Type Culture Collection) were cultured in EMEM containing 10% fetal bovine serum and 1× Pen/Strep. Opsin coding sequences were codon optimized for mammalian usage (Entelechon and Geneart), cloned into pcDNA3.1 with and without 1D4 antigen tag. Point mutations were introduced using QuikChange Site Directed Mutagenesis Kit (Strategene). Receptor activation by light was assayed employing an impedance-based Real-Time Cell Analysis (RTCA) System (Roche Applied Science). HEK and N2A cells (passage 6–8) were seeded on a 96-well E-plate (Roche) at a cell density of 35,000 and 10,000 cells/well, respectively. Eighteen to 24 h later, cells were transfected using TransIT-LT1 (Mirus Bio) at a DNA–transfection reagent ratio of 1∶3 according to the manual. Cells were supplied with 50 µM 11-cis-retinal (a generous gift from Rosalie Crouch and the National Eye Institute, USA) to assure proper receptor reconstitution. Receptor activation was assayed repeatedly during a time window of 24–56 h after transfection by exposing the cells for 10 min to white light (LED, spectrum: 450 nm<λ<750 nm, 1.3×10^16^ photons/cm^2^/s) or to near infrared (NIR) light (λ = 950 nm).

The RTCA system detects changes in cellular morphology (and possibly also channel conductance) elicited by surface receptor activation, which are reflected in changes of the CI value (Figure S13A) [23]. CI values were collected every 15 s, normalized by dividing the CI at the time point before light onset (Figure S13B), and baseline-corrected by subtracting the CI obtained under non-light-sensitive conditions (human Oxytocin receptor-transfected) (Figure S13C). The cell response was quantified as area under the curve (AUC) (Figure S13D) in Matlab, and data were analyzed by the Mann–Whitney Test using the GraphPad Prism software.

### Wavelength Sensitivity Assay

Wavelength sensitivity assay was performed using the RTCA DP station (Figure S4D,E), which allowed for measurement of three individual and separated 16-well E-plates at the same time. A black box with windows facilitated the illumination of the three plates with differently filtered light (Lee filter; blue, no. 071; red, no. 787; green, no. 736) while at the same time preventing light from leaking from one plate to the other. Light intensities were adjusted with neutral density filters (Lee filters) and measured with a USB2000+ spectrometer (Ocean Optics; blue, 3.6×10^14^ photons/cm^2^/s; green, 1.9×10^14^ photons/cm^2^/s; red, 7.4×10^14^ photons/cm^2^/s). Comparison of receptor activation to different light colors required standardization of cell responses to white light. Cells were therefore exposed to a 10 min white light pulse 60 min prior to the color light pulse (Figure S4C). AUC during the color light pulse was normalized to AUC of the preceding white light pulse and plotted as Normalized Response (%AUC). Quantification and statistical tests as described above.

### Whole Mount in Situ Hybridization on Larvae

WMISH on 7 dpf medaka were performed as described before [54] with modifications: 25 min 10 µg/ml Proteinase K, (pre-)hybridization at 65°C, posthybridization washes at 65°C twice for 30 min with 50% formamide/2× SSCT, 15 min with 2× SSCT, and twice 30 min with 0.2× SSCT. Embryos were blocked in 5% sheep serum in 1× PTW (PBS+0.1% Tween-20) and subsequently incubated in a 1∶2,000 dilution of alkaline phosphatase-coupled anti-digoxigenin antibody (Roche) in 2.5% sheep serum/1× PTW, and staining proceeded in NBT/BCIP staining buffer containing 10% Polyvenyl alcohol (w/v). WMISH on zebrafish [5] as well as two-color ISH were done as described before [55]. Pictures were taken on a Zeiss Axioplan 2.

### In Situ Hybridization on Adult Brain Sections

Adult fish were anesthetized in fish water containing 0.2% tricaine, decapitated, and the brain dissected, subsequently fixed in ice-cold 4% PFA overnight. ISH staining on adult brain sections (AB strain) were described before [56]. For two-color ISH, the protocol for whole mount larvae (above) was applied to sections. Pictures were taken on a Zeiss Axioplan 2 and a Leica MZ 16 FA.

### Antibodies and Immunohistochemistry

Polyclonal antibodies against the intracellular C-terminus of medaka TMT-1B (_305_NKQFYRCFWAFFCCSTPEQVSTLRTFSRVTKTIRTFRQERELHVSAPAPSSGLPTPNSIQKGNNHVDPSSINQACAASDSPDSRKPKVVLVAHYQE_400_) were raised commercially by injection of a His-tagged version of the peptide into rabbit (Primm Srl). Serum from immunized rabbits was affinity purified (Primm srl), diluted 1∶1 with glycerol, and used at a 1∶250 dilution. Pre-immune serum from the same rabbit (1∶100) was used as control, as well as antibody pre-incubated with a 5-fold excess (by weight) of recombinant peptide.

The ChAT antibody (Merck Millipore, AB144P) has already previously been used successfully to describe the cholinergic system in a variety of nonmammalian vertebrate species including the teleosts trout [57] and zebrafish [35] and used at a 1∶100 dilution.

For immunohistochemical stainings, brains were fixed in 4% paraformaldehyde in PBT (PBS with 0.1% Triton X-100) for 2 h, embedded in 3% agarose, and cut into 100 µm sections on a vibratome. Blocking was performed in 5% natural donkey serum (NDS). The Ola-TMT-1b and ChAT antibodies were incubated for 3 d in 1% NDS. After PBT washes, sections were treated with secondary antibodies (1∶200; donkey anti-rabbit-Cy3, 711-165-152 and donkey anti-goat-Alexa488, 705-545-147; Jackson ImmunoResearch) for 3 d, washed with PBT, and mounted in DABCO-glycerol. Pictures were taken on a Zeiss LSM710 confocal scanning microscope and noise corrected using FIJI [58].

### Brain Slice Preparation

Adult medaka fish were anesthetized in fish water containing 0.2% tricaine and decapitated. Heads were immersed in ice-cold oxygenated (95% O_2_/5% CO_2_) dissection solution containing (in mM) 110 choline chloride, 25 NaHCO_3_, 1.25 NaH_2_PO_4_, 2.5 KCl, 0.5 CaCl_2_, 7 MgCl_2_, 11.6 ascorbic acid, 3.1 pyruvic acid, and 25 D-glucose (final pH = 7.4). After dissection, the brains were embedded in 2% low-melting agarose (Sigma), and acute coronal whole-brain slices (180 µm) were made using a vibratome (leica VT1200S, Germany) at high vibration frequency. Slices were transferred to a resting chamber filled with standard artificial cerebrospinal fluid (ACSF) composed of (in mM) 118 NaCl, 2.5 KCl, 26.5 NaHCO_3_, 1 NaH_2_PO_4_, 1 MgCl_2_, 2 CaCl_2_, and 20 D-glucose; aerated constantly with 95% O_2_/5% CO_2_; and maintained at room temperature throughout the experiments.

### Electrophysiology

Slices were transferred to a submerged recording chamber and immobilized by a silver grid with attached nylon mesh (Figure S11A). Individual interneurons located ventrally along the stratum periventriculare were visualized with an upright microscope (Olympus BX51W1, Germany) equipped with infrared video microscopy and differential contrast optics. Whole-cell patch-clamp recordings in the current-clamp (<30 MΩ access resistance) mode were performed using borosilicate pipettes pulled on a Flaming/Brown micropipette puller (Sutter Instrument, Novato, CA), yielding a final resistance of 4–5 MΩ, and filled with an intracellular solution containing (in mM) 130 K.gluconate, 5 KCl, 2.5 MgCl_2_, 10 HEPES, 0.6 EGTA, 4 Na_2_ATP, 0.4 Na_3_GTP, and 10 Na_2_-phosphocreatine (pH = 7.25, adjusted with KOH; 290 mOsm). Data were acquired from the soma of individual interneurons with a Multiclamp 700B amplifier (Axon Instruments, Molecular Devices, Foster City, CA), low-passed filtered using the 10 kHz four-pole Bessel filter, sampled at 10 kHz (Digidata 1440A, Axon Instruments), and collected using pClamp10 software (Molecular Devices, Inc., USA). Measurements were not corrected for the liquid junction potential. Offline analysis was made using the data analysis software Clampfit 10.2 (Molecular Devices, Inc., USA). Care was taken to record from individual interneurons ventrally located in different positions along the optic tectum (Figure S11). The slices were continually superfused in a closed circuit with ACSF oxygenated with 95% O_2_/5% CO_2_ and with 50 µM 11-cis-retinal.

### Characterization of Light-Induced Neuronal Responses in the Optic Tectum

All recordings were made in complete darkness, except during the light stimulation episode. After reaching whole-cell configuration, neurons were kept in darkness for an initial period of 2 min, followed by a 1-min light stimulation and a further recovery period of 2 min darkness. Light stimulation was generated with a standard halogen lamp-source (2.53×10^16^ photons/cm^2^/s) and delivered via an optic fiber positioned directly adjacent to the recording area. The timing and duration of the light stimuli were controlled via a shutter driver (model VCM-D1, UniBlitz, USA) and triggered using Clampex 10.2 software. Consecutive recordings among individual interneurons were interleaved by a 20-min minimum period of darkness. Light intensity was measured using an optical power meter (Ocean Optics).

### Data Analysis

The magnitude of the responses (defined as a variation in membrane potential) was assessed by subtracting the absolute value (in mV), taken immediately before the light stimulation (time = 0), to the mean value (in mV) across a 1-min time-window placed along the last dark minute before the light stimulation (time = −1), during the light stimulation (time = 1), and along the first dark minute after light cessation (time = 2). The significance of the differences between the magnitude of the responses across the different time points was evaluated by the paired Student's *t* test.

### Genbank Accession Numbers

Cloned sequences reported in this article were deposited in GenBank (Table S1).

## Supporting Information

Figure S1 A phylogenetic analysis of the ETO family places the opossum TMT-Opsin within the TMT-Opsin 2 group. Maximum likelihood tree of vertebrate TMT-Opsins and Encephalopsins using Rhodopsins as outgroup reveals conservation of TMT-Opsin 2 up to marsupials. Phylogenetic tree was calculated on the phylogeny.fr platform (http://www.phylogeny.fr) [59]. Protein sequences were first aligned with MUSCLE [60], alignments were checked for accuracy with G-blocks [61], and PhyML was used for tree building (maximum-likelihood method) [62]. The numbers at the nodes are percentage and indicate the bootstrap values obtained from 500 bootstrap replicates. Trees were rendered using Treedyn [59] and Mega. The following protein sequences were used: Rhodopsin (*O. latipes*, NP_001098165.1; *D. rerio*, P35359.2; *B. taurus*, NP_001014890.1; *M. domestica*, XP_001366225.1; *M. musculus*, NP_663358.1; *H. sapien*, NP_000530.1; *G. gallus*, NP_001025777.1), Encephalopsin (*H. sapien*, NP_055137.2; *M. musculus*, NP_034228.1; *G. gallus*, XP_426139.2; *O. latipes*, JX293359; *D. rerio*, ABM65699), TMT-Opsin 1 (*O. latipes*, JX293354, JX293355; *D. rerio*, AAL83431, JX293360; *P. andruzzii*, ADL62693.1), TMT-Opsin 2 (*O. latipes*, JX293356; *D. rerio*, JX293361, JX293362; *G. gallus*, JX293365; *M. domestica*, JX293366), and TMT-Opsin 3 (*O. latipes*, JX293357; *D. rerio*, JX293363, JX293364).
(EPS)

Figure S2 Verification of recombinant TMT-Opsin expression in HEK293 cells by immunocytochemistry. Immunocytochemical detection of TMT-Opsins recombinantly expressed in HEK293 cells and co-stained with DAPI. Note the correct expression of all four medaka TMT-Opsins at the membrane. Scale bars, 50 µm.
(TIF)

Figure S3 Group 2 TMT-Opsins exhibit different response kinetics than group 1 and 3 TMT-Opsins. Results were obtained using opsin-transfected N2A cells and measuring changes in cell impedance upon light stimulation with a real-time cell analysis (RTCA) system. Cell impedance was displayed as CI values. CI values were normalized to CI values just prior to light onset and were baseline-corrected by substracting CI traces obtained from cells transfected with the light insensitive human Oxytocin receptor. Data are presented as mean (*N* = 4). (A) Comparison of medaka (Ola) and zebrafish (Dre) group 2 TMT-opsins. We compared three different experiments (E1, E2, and E3), each with four separate light pulses (L1–L4). Responses to each light pulse were plotted into the same graph for all three experiments. Note the similarity of the response shape between different homologues/paralogues of zebrafish and medaka fish. (B) Comparison of medaka TMT-Opsin 2 to group 1 and 3 TMT-Opsins of medaka (Ola TMT-1B and 3A) and zebrafish (Dre TMT-1B). The same three experiments as in (A) were compared. Again, responses to each light pulse were plotted into the same graph for all three experiments. Note the obvious difference in response kinetics between TMT-Opsin 2 and other TMT-Opsins: TMT-Opsin 2 response curves drastically drop after the light was turned off, while other TMT-Opsin responses slowly decreased.
(EPS)

Figure S4 Irradiance curves for differently filtered light and experiment setup for assessment of the absorption maxima. A light-emitting diode (LED) source was used to produce white light (400 nm<λ<800 nm). (A) Spectral profile of color filters used to color light: blue (λ_peak_ = 450 nm), green (λ_peak_ = 528 nm), and red (λ_max. peak_ = 605 nm). (B) Magnification of dashed box of spectral profile in (A). The light intensity was adjusted with neutral density filters in order to reach a similar activation strength of human rhodopsin to both the blue and the green filter, thus calibrating the setup to the human rhodopsin absorption maximum of 497 nm (dashed line). Note that the absolute intensities are also within a similar range (blue, 3.6×10^14^; green, 1.9×10^14^; red, 7.4×10^14^). (C) Example traces of one example wavelength sensitivity experiment. Wavelength sensitivity assay was performed using the RTCA DP station, which allowed for measurement of three individual and separated cell culture plates at the same time. A black box with windows facilitated the illumination of the three plates with differently filtered light (E). Comparison of receptor activation to different light colors required standardization of cell responses to white light. Therefore, cells were first exposed to a 10 min white light pulse (D). Sixty minutes later, the same cells were being exposed to light of different colors (E). Note the stronger response of cells, which were later exposed to green light in this particular example. By assessing cell responses to white light first and then comparing it to the response to color light, we could minimize variances between plates within one experiment. Dashed CI traces show cell responses to white light, and solid traces show responses to filtered light.
(EPS)

Figure S5 TMT-Opsins and Encephalopsin do not respond to near infrared (NIR) light. Results were obtained using opsin-transfected HEK293 (HEK) cells and measuring changes in cell impedance upon light stimulation with a real-time cell analysis (RTCA) system. Cell impedance was displayed as CI values. CI values were normalized to CI values just prior to light onset and were baseline-corrected by substracting CI traces obtained from cells transfected with a light insensitive Oxytocin receptor. (A–D) HEK cells transfected with native medaka TMT-Opsins or Encephalopsin (blue) were compared to mutated (L294A) opsins (black). The light stimulation was always 10 min (yellow background box). No change in cell impedance could be detected when cells were exposed to NIR light. Data are presented as mean ± SEM (*N* = 12). (F) Quantification of opsin-dependent cell responses. Relative light responses are displayed as mean ± SEM (*N* = 12). ns, not significant.
(EPS)

Figure S6 Light-dependent opsin activation in HEK cells causes a characteristic shape of cell impedance traces. (A–C) Traces of HEK cells transfected with native medaka TMT-Opsins (red) were compared to mutated (L294A) TMT-Opsins (black) during a 10 min light stimulation (yellow background box) phase. TMT-Opsin activation evokes a typical light-dependent CI change compared to the mutated version. Note the characteristic shape of the curves marked by an abrupt drop of cell impedance right after light onset followed by a steep ascent of the cell impedance. Data represent mean ± SEM (grey lines) (*N* = 32).
(EPS)

Figure S7 MT-Opsins are specifically expressed in light sensory organs and brain areas suspected to harbor deep brain photoreceptors in medaka fish. Results were obtained using ISH. Scale bars, 100 µm. (A–D) Whole-mount ISH on 7 dpf medaka larvae reveal TMT-Opsins expression in dedicated light sensory organs. (A) Dorsal view of *tmtops2* expression in distinct areas of the anterior nervous system. No expression could be detected in peripheral tissues. (B) Side view of *tmtops1b* expression in amacrine cells of the eye. (C) Side view of *tmtops2* expression in amacrine cells (white arrowheads), the iris (black arrowhead), and the annular ligament (red arrowhead) of the eye. (D) Dorsal view of pineal *tmtops2* expression. Higher magnification of the pineal is shown in the inset. (E–G) ISH on sections of adult brain identify TMT-Opsin expression in deep brain photoreceptor brain areas. (E) Coronal section showing expression of *tmtops2* in the ventromedial thalamic nucleus (VM) and the preoptic area (PP) close to the ventricle (v). (F) The same expression areas can be seen on the sagittal section cut close to the midline of the brain. The dashed line indicates the transversal plane corresponding to sections in (E) and (G). (G) Coronal section showing expression of *tmtops1b* in the anterior thalamic nucleus (A).
(TIF)

Figure S8 Sense RNA controls for tested TMT-Opsins. ISH controls done with sense RNA probes of all tested medaka TMT-Opsins on coronal adult brain sections. No specific staining could be detected in any of the sense controls.
(TIF)

Figure S9 Controls for specificity of the polyclonal rabbit anti-TMTopsin1b antibody. (A–F) Immunocytochemical staining of TMTopsin1b-1D4 expressing N2A cells co-stained with DAPI (Methods S1). (A–B) Co-staining with the polyclonal rabbit anti-TMTopsin1b antibody (A) and the anti-1D4 antibody (B). Note the clear co-labeling of membrane bound receptor. (C–F) Antibody specificity was verified by staining with pre-immune serum (C), by omitting the primary antibody (D), by staining with a peptide-neutralized antibody (E), and by staining of untransfected cells (F). (G–J) Antibody specificity was further assessed by staining of brain sections with peptide-neutralized antibody (G–H) or the pre-immune serum (I–J). No specific staining could be detected with both controls, neither in the hindbrain (G, I), nor in the dorsal tegmental nucleus (H, J). Scale bars 100 µm.
(TIF)

Figure S10 *Tmtops2* and *valop* are co-expressed in inter- and motorneurons. Two-color ISH of *tmtops2* (blue) and *chat1/2* (red/red fluorescence) on coronal adult medaka brain sections. (B, E) Magnification of boxed areas. (C, F) Fluorescent images of *chat1*/*2* staining. Arrowheads, co-expressing cells. Scale bars, 50 µm. Co-staining in facial nerve motorneuron (A–C) and in interneurons of the periventricular grey zone of the tectum (D–F). Note the clear presence of blue and red signal in the same cell of the facial nerve nucleus (B, C).
(TIF)

Figure S11 Whole-cell patch-clamp setup for medaka brain slices. IR-DIC images from a coronal whole-brain slice depicting the optic tectum (OT) as well as the targeted interneuron ventrally located along the stratum periventriculare (SPV).
(TIF)

Figure S12 *Tmt-opsin* expression in zebrafish reveals evolutionary conservation of expression domains. ISH on 6 dpf zebrafish larvae (A, B) and coronal sections of the adult brain (C–I). Scale bars, 100 µm. (A) *tmtops2a* expression in amacrine cells and the annular ligament (black arrowhead). Pineal expression of *tmtops2a* in larval (B) and adult brains (C). *tmtops2a* in the facial nerve nucleus (D) and the dorsal tegmental nucleus (E). The dorsal tegmental nucleus was also stained for *tmtops3a* (F) and *tmtops1b* (G). *tmtops1b* is also present in the facial nerve nucleus (H) and the central posterior thalamic nucleus (I).
(TIF)

Figure S13 Normalization, baseline-correction, and quantification of impedance responses. (A) CI traces during light exposure. (B) For analysis, CI values were normalized by dividing by the CI at the last time point before light onset. (C) In order to correct for changes unrelated to the light response, the baseline (Oxytocin receptor) was subtracted from all traces. (D) Quantification was obtained by dividing the area under the curve (AUC) during 10 min light exposure by AUC during the preceding 10 min dark phase and given as relative light response (AUC) in Figure 2F,I.
(EPS)

Methods S1 Immunocytochemisty.
(DOCX)

Table S1 Accession numbers and PCR primers. Summary of the GenBank accession numbers for each of the *D. rerio*, *O. latipes*, and *Gallus gallus* cloned and sequenced cDNAs including sequences of forward and reverse PCR primers as well as nested primers used to amplify ETO genes. Summary of Ensemble coordinates for predicted opsins used in Figure 1A.
(PDF)

## Abbreviations

AUC: area under the curve
CI: cell index
dpf: days postfertilization
CSF-contacting: central spinal fluid-contacting
*Dre*: *Danio rerio*
ETO: Encephalopsin-TMT-Opsin phylogenetic group
GPCR: G-protein-coupled receptor
ISH: in situ hybridization
N2A: Neuro-2A cells
NIR: near infrared
*Ola*: *Oryzias latipes*
RTCA: Real-Time Cell Analysis Assay
TMT-Opsin: teleost-multiple-tissue opsin
VAL-Opsin: vertebration ancient long opsin
