# Supplementary figures and images for: Co-Expression of VAL- and TMT-Opsins Uncovers Ancient Photosensory Interneurons and Motorneurons in the Vertebrate Brain
Source: PLoS Biol. 2013 Jun 11;11(6):e1001585. doi: 10.1371/journal.pbio.1001585 (PMC3679003; doi:10.1371/journal.pbio.1001585)

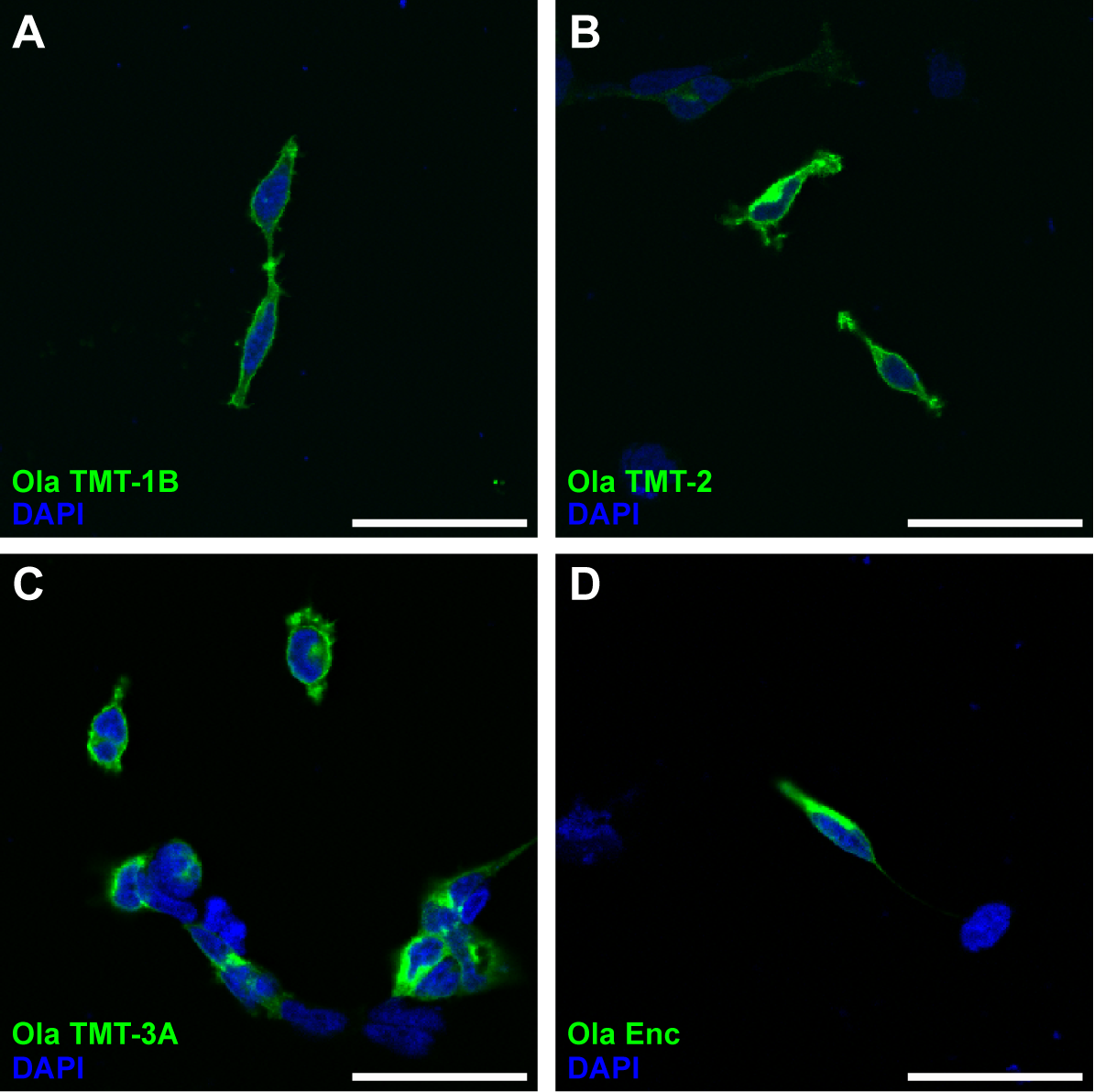

Supplement: Figure S2 — Verification of recombinant TMT-Opsin expression in HEK293 cells by immunocytochemistry. Immunocytochemical detection of TMT-Opsins recombinantly expressed in HEK293 cells and co-stained with DAPI. Note the correct expression of all four medaka TMT-Opsins at the membrane. Scale bars, 50 µm. (TIF) [file pbio.1001585.s002.tif]

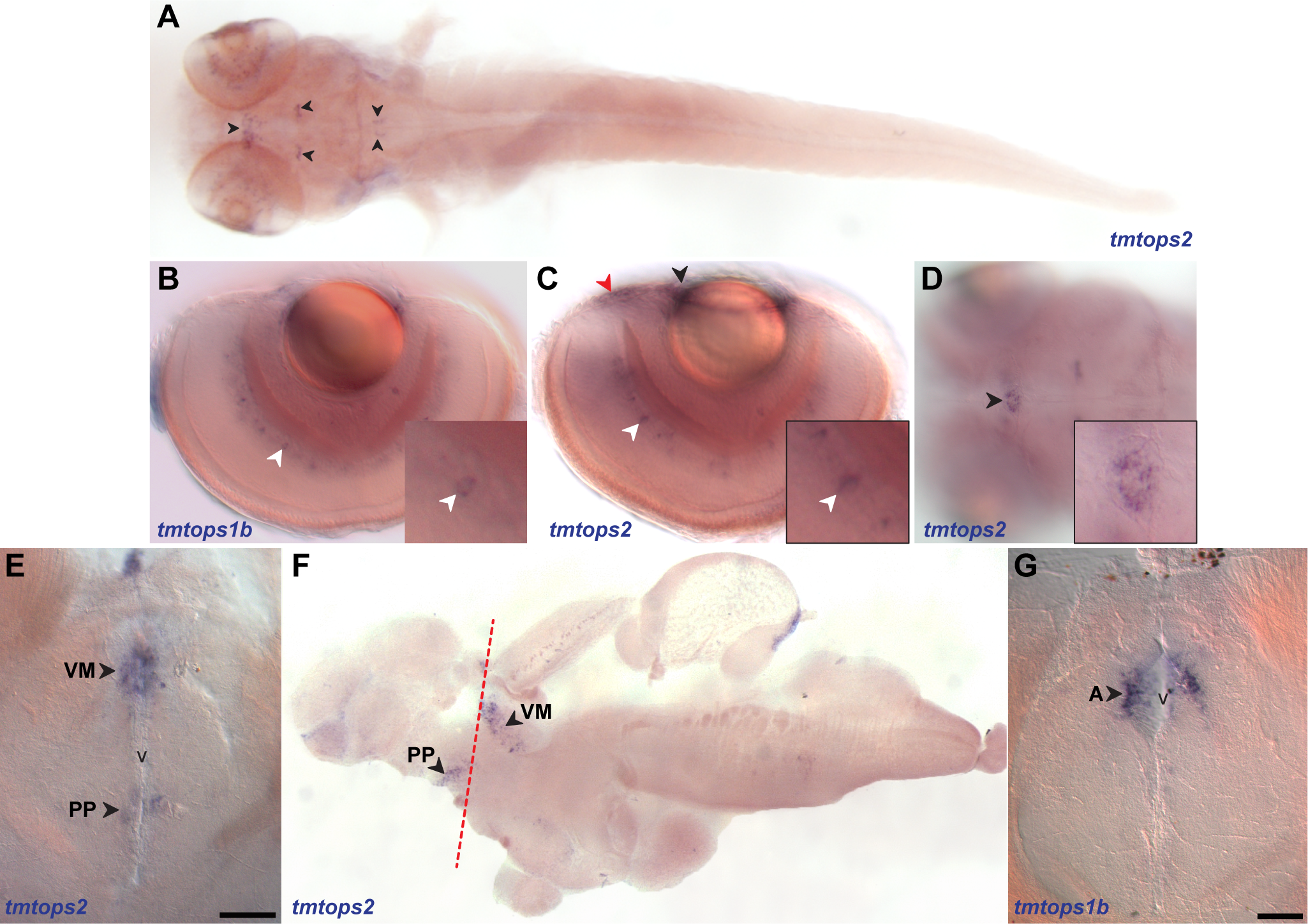

Supplement: Figure S7 — MT-Opsins are specifically expressed in light sensory organs and brain areas suspected to harbor deep brain photoreceptors in medaka fish. Results were obtained using ISH. Scale bars, 100 µm. (A–D) Whole-mount ISH on 7 dpf medaka larvae reveal TMT-Opsins expression in dedicated light sensory organs. (A) Dorsal view of tmtops2 expression in distinct areas of the anterior nervous system. No expression could be detected in peripheral tissues. (B) Side view of tmtops1b expression in amacrine cells of the eye. (C) Side view of tmtops2 expression in amacrine cells (white arrowheads), the iris (black arrowhead), and the annular ligament (red arrowhead) of the eye. (D) Dorsal view of pineal tmtops2 expression. Higher magnification of the pineal is shown in the inset. (E–G) ISH on sections of adult brain identify TMT-Opsin expression in deep brain photoreceptor brain areas. (E) Coronal section showing expression of tmtops2 in the ventromedial thalamic nucleus (VM) and the preoptic area (PP) close to the ventricle (v). (F) The same expression areas can be seen on the sagittal section cut close to the midline of the brain. The dashed line indicates the transversal plane corresponding to sections in (E) and (G). (G) Coronal section showing expression of tmtops1b in the anterior thalamic nucleus (A). (TIF) [file pbio.1001585.s007.tif]

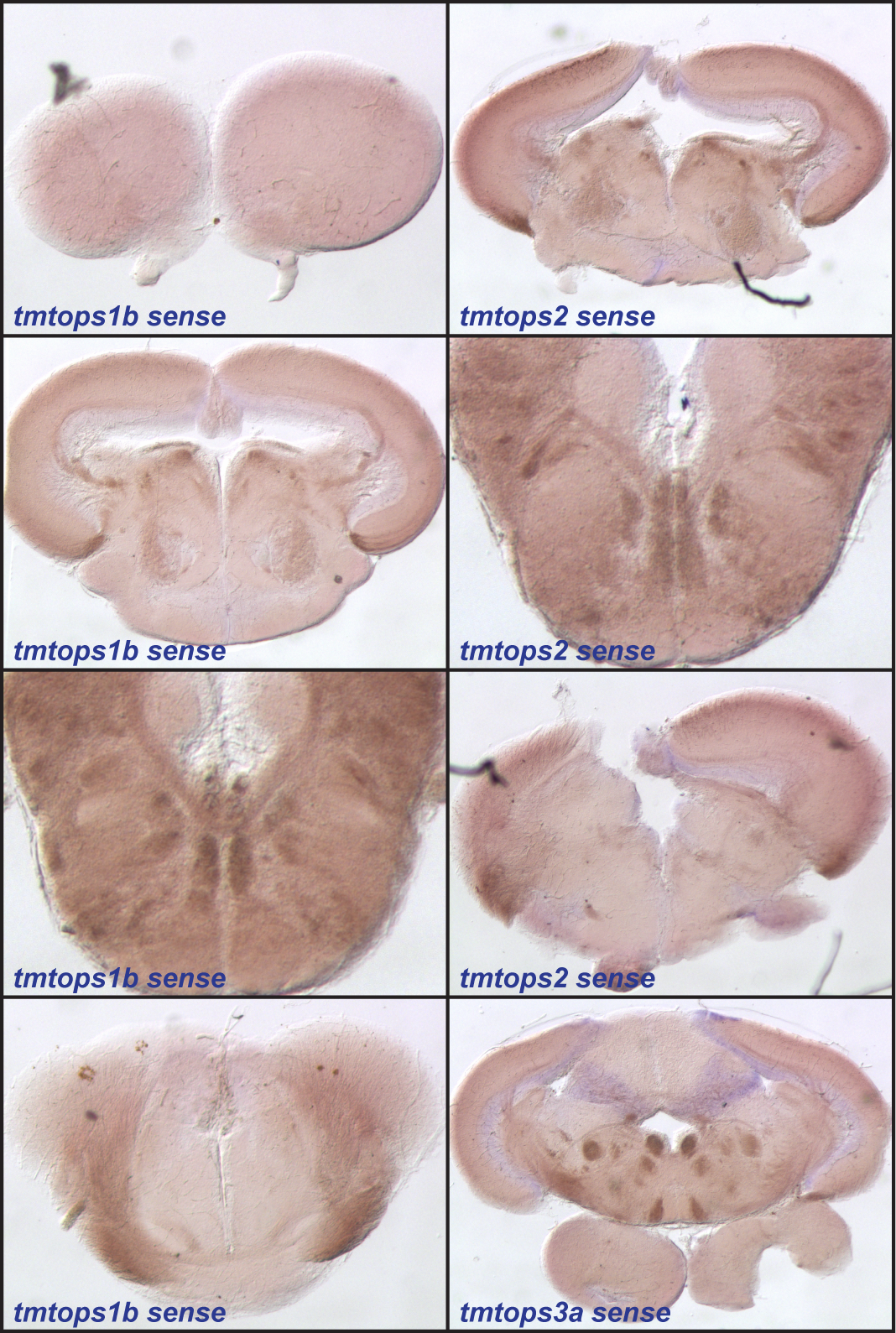

Supplement: Figure S8 — Sense RNA controls for tested TMT-Opsins. ISH controls done with sense RNA probes of all tested medaka TMT-Opsins on coronal adult brain sections. No specific staining could be detected in any of the sense controls. (TIF) [file pbio.1001585.s008.tif]

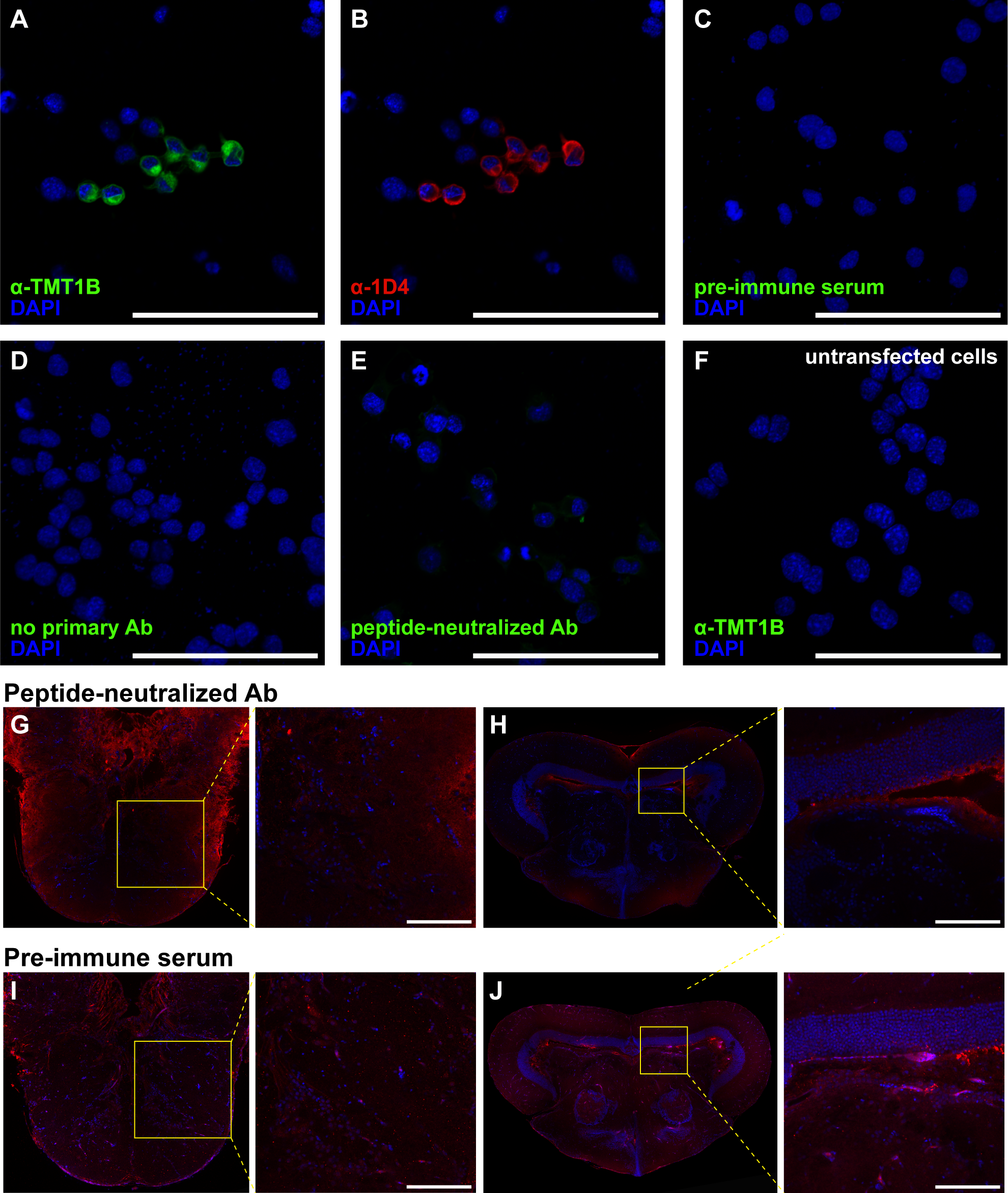

Supplement: Figure S9 — Controls for specificity of the polyclonal rabbit anti-TMTopsin1b antibody. (A–F) Immunocytochemical staining of TMTopsin1b-1D4 expressing N2A cells co-stained with DAPI (Methods S1). (A–B) Co-staining with the polyclonal rabbit anti-TMTopsin1b antibody (A) and the anti-1D4 antibody (B). Note the clear co-labeling of membrane bound receptor. (C–F) Antibody specificity was verified by staining with pre-immune serum (C), by omitting the primary antibody (D), by staining with a peptide-neutralized antibody (E), and by staining of untransfected cells (F). (G–J) Antibody specificity was further assessed by staining of brain sections with peptide-neutralized antibody (G–H) or the pre-immune serum (I–J). No specific staining could be detected with both controls, neither in the hindbrain (G, I), nor in the dorsal tegmental nucleus (H, J). Scale bars 100 µm. (TIF) [file pbio.1001585.s009.tif]

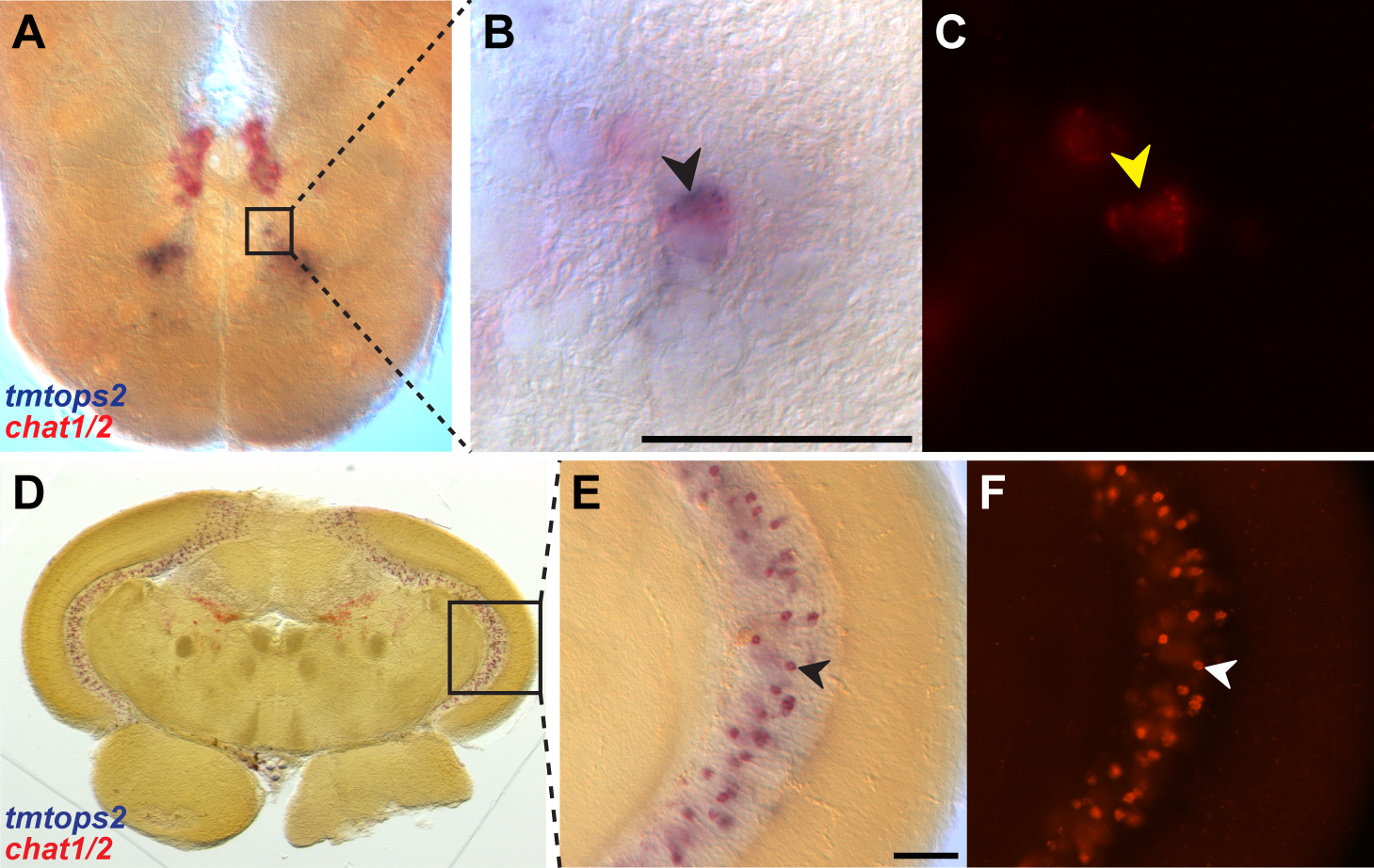

Supplement: Figure S10 — Tmtops2 and valop are co-expressed in inter- and motorneurons. Two-color ISH of tmtops2 (blue) and chat1/2 (red/red fluorescence) on coronal adult medaka brain sections. (B, E) Magnification of boxed areas. (C, F) Fluorescent images of chat1/2 staining. Arrowheads, co-expressing cells. Scale bars, 50 µm. Co-staining in facial nerve motorneuron (A–C) and in interneurons of the periventricular grey zone of the tectum (D–F). Note the clear presence of blue and red signal in the same cell of the facial nerve nucleus (B, C). (TIF) [file pbio.1001585.s010.tif]

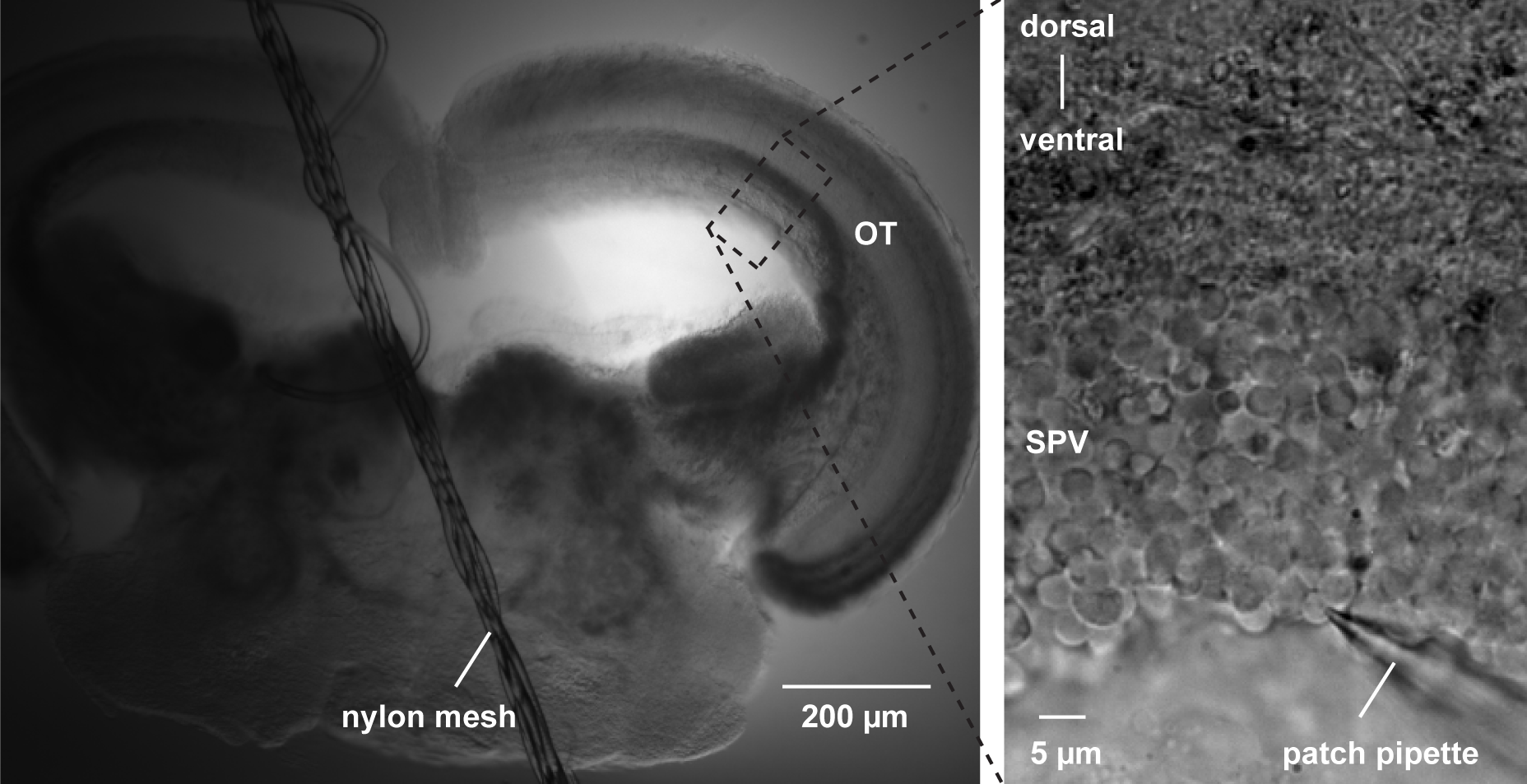

Supplement: Figure S11 — Whole-cell patch-clamp setup for medaka brain slices. IR-DIC images from a coronal whole-brain slice depicting the optic tectum (OT) as well as the targeted interneuron ventrally located along the stratum periventriculare (SPV). (TIF) [file pbio.1001585.s011.tif]

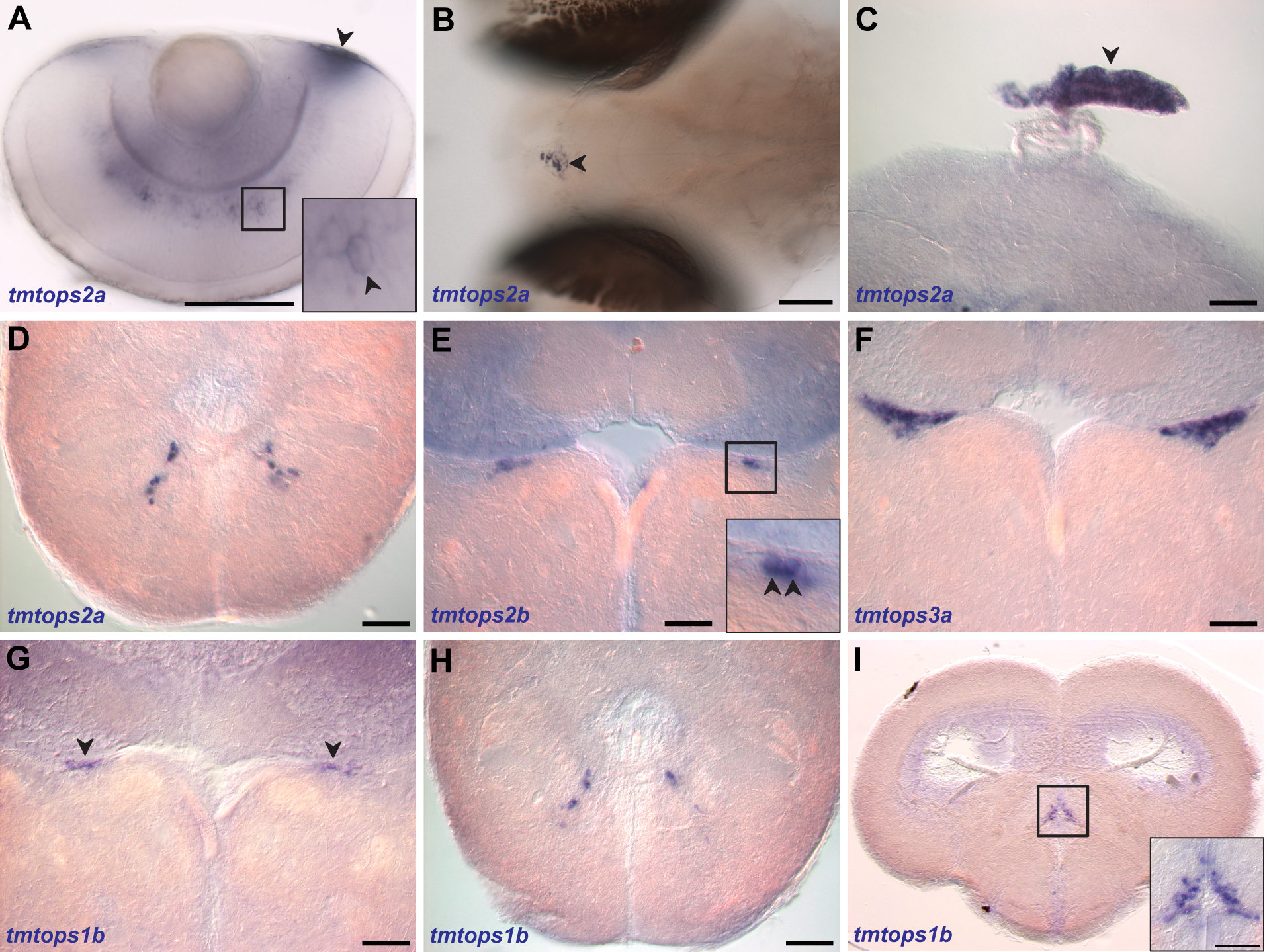

Supplement: Figure S12 — Tmt-opsin expression in zebrafish reveals evolutionary conservation of expression domains. ISH on 6 dpf zebrafish larvae (A, B) and coronal sections of the adult brain (C–I). Scale bars, 100 µm. (A) tmtops2a expression in amacrine cells and the annular ligament (black arrowhead). Pineal expression of tmtops2a in larval (B) and adult brains (C). tmtops2a in the facial nerve nucleus (D) and the dorsal tegmental nucleus (E). The dorsal tegmental nucleus was also stained for tmtops3a (F) and tmtops1b (G). tmtops1b is also present in the facial nerve nucleus (H) and the central posterior thalamic nucleus (I). (TIF) [file pbio.1001585.s012.tif]
