# Supplementary material for: Co-Expression of VAL- and TMT-Opsins Uncovers Ancient Photosensory Interneurons and Motorneurons in the Vertebrate Brain
Source: PLoS Biol. 2013 Jun 11;11(6):e1001585. doi: 10.1371/journal.pbio.1001585 (PMC3679003; doi:10.1371/journal.pbio.1001585)
Supplement: Methods S1 — Immunocytochemisty. (DOCX) [file pbio.1001585.s014.docx]

## Supporting Information Materials and Methods

### Immunocytochemisty

HEK and N2A cells were grown on Poly-D-Lysine coated coverslips and transfected with plasmids encoding different TMT-Opsins fused to a c-terminal 1D4 antigen tag. 24 hours after transfection, cells were fixed with 4% formaldehyde in PBS, permeabilized and blocked with blocking buffer (10% sheep serum/0.1% Triton X-100 in PBS). Cells were incubated either with primary mouse anti-1D4 antibody (Sigma, R5403, 1:100) or rabbit anti-TMTopsin1b (1:250) or pre-immune serum (1:250) in blocking buffer for 2 hours. The secondary Alexa488 or Cy3 coupled goat anti-mouse or anti-rabbit antibodies (Invitrogen) were diluted 1:500 in 0.2% BSA/0.1% Triton X-100 with DAPI in PBS and cells incubated for 1hr. Finally, cells were mounted in Prolong Gold containing and imaged on a confocal microscope.
