# Supplementary material for: Co-Expression of VAL- and TMT-Opsins Uncovers Ancient Photosensory Interneurons and Motorneurons in the Vertebrate Brain
Source: PLoS Biol. 2013 Jun 11;11(6):e1001585. doi: 10.1371/journal.pbio.1001585 (PMC3679003; doi:10.1371/journal.pbio.1001585)
Supplement: Table S1 — Accession numbers and PCR primers. Summary of the GenBank accession numbers for each of the D. rerio, O. latipes, and Gallus gallus cloned and sequenced cDNAs including sequences of forward and reverse PCR primers as well as nested primers used to amplify ETO genes. Summary of Ensemble coordinates for predicted opsins used in Figure 1A. (PDF) [file pbio.1001585.s015.pdf]

Table S1: Accession numbers and corresponding PCR primers.

Oranism	Gene name	Accession number	forward primer	reverse primer	forward nested primer	reverse nested primer
O. latipes	*TMT-opsin 1A*	JX293354	ACCGCAGTGACACGGACT	TGATGCTCCCCTCTCATTGT	ATGCTGGTGTCCAACGTGAG	AATTGTCAGCCATCAAAGTGG
O. latipes	*TMT-opsin 1B*	JX293355	GTCCTCAGTTGGCGGCTCT	GCCTTCTGCTTTTAGGGGATT	GGCATGATCGTTCCCAAC	TTTCCTCATTCTTTCACTCCTGA
O. latipes	*TMT-opsin 2*	JX293356	AGGACAAAGTCAGATGAATGGA	TTCAGATAAGTCGCGCTCAC	GGATGTTTTCCGGTCAGACT	GGAACACCTAGTTGGCGTTATC
O. latipes	*TMT-opsin 3A*	JX293357	TTGATGCACCTCTCTGTTGG	GCATGCTCATGGTCAGGTT	CCTGAATGATATGGTCCTCCA	TTTATGGGGTGTAGTGGACAAA
O. latipes	*TMT-opsin 3B*	JX293358	AAACACCTGGAAAACTGGTCGT	AAACACCTGGAAAACTGGTCGT		
O. latipes	*Encephalopsin*	JX293359	ACAACTTTTCCTTCTCGCC	CCGGAGTTGCATGTTTGGTT	ATGAATCCAGCAAACGAAAGC	TCACAATGGCCTCACGTG
O. latipes	*VAL-opsin*	AB383148	ATGGATTCGTTGAGCTTGTCA	TGGACACACTTTGTTCTCTGG		
D. rerio	*TMT-opsin 1A*	AAL83431	TGCTCAATGGATTGCGGATTGCG	TAGGTTTGCCTGACTGTACAACC	ATGATTGTGTCCAACTTGAGTGTG	TCATCCGTTATAATGGGCCACGAG
D. rerio	*TMT-opsin 1B*	JX293360	CACAAGCCATAATTAATGGTGCA	TAGGTTTGCCTGACTGTACAACC	ATGATTGAGTCTAACGTGAGTCG	TCAGCCATTGTAATAGGCCACAAG
D. rerio	*TMT-opsin 2A*	JX293361	CGCAGTAAACGTAACATCAGTTG	AGGCGAAGCTTTGTGGACT	CGGCAAAGATGTTTCCTGA	TTAGCCAGAGACAGGGGTGCT
D. rerio	*TMT-opsin 2B*	JX293362	GACTCGAACGAACTTTTTTAGCT	GCATTCAGCCCTGGTCAGTTTTCT	ATGTTTTTCGAGCAGGCCGATTTAAAC	TAAATGTCAATGTTCATGGAATG
D. rerio	*TMT-opsin 3A*	JX293363	CAACATCATTTCCCCGTGCAGG	CTAAAAGCTGGTCTGTAAGATCATGG	ATGGTCGTCTACATCTGGAGTTTGAA	TCAGGGTGTGTAGTGAACCACCAAG
D. rerio	*TMT-opsin 3B*	JX293364	CTTCTCTATTAGTGTGCTGCTTC	TCATATGGTTTATCATGGATTGAACT	CCAGTAGTATGGTCACTGTCC	TGCAGGGCTTTTATGGAGTAC
D. rerio	*Encephalopsin*	ABM65699	CCATGTTGGGAGTTGACTTGGAA	TTAATGTCTCCACGGCAAGTG	ATGAATTCTTTTAATGAAACACCGAC	TCAAAGCGGTCGAACTTGAATTACG
G. gallus	*TMT-opsin 2*	JX293365	CCTGTTGAGCCAAGAGCTGG	TGGGATTGCAAACAGTGCTGC	CTGTCACGAAATGGGCACAC	GCTAAGATGGAAGGTATGATGC
G. gallus	*Encephalopsin*	XP_426139.2	ATGCACTCGGGGAACGGC	CTATAGTGGTTTTACTTGGATGAC	AGCGTCCGCTCTTCAGCG	CTTTTGTCCCATTGTGTTTGCTG
O. latipes	*ChAT1*	ENSORLG00000006943	GCTCACGGAAGAGCAATTCA	AGTGTCGGACCTTTCCTTCG		
O. latipes	*ChAT2*	ENSORLG00000009452	TGACTACTGGCTGGAGGACA	CTGTCAGCCGGTTTGGATAC		
M. domestica	*TMT-Opsin 2*	JX293366				

Oranism	Gene name	Chromosome	Predicted exons in Ensemble				
T. nigroviridis	*TMT-opsin3-like*	1	9073146 - 9073524	9072296 - 9072624	9071002 - 9071235	9070809 - 9070874	
T. nigroviridis	*Opn3-like*	UnRandom	53814107 - 53814426	53790241 - 53790560	53788487 - 53788738	53788009 - 53788230	
T. rubripes	*TMT-opsin1-like*	scaffold 263	36143 - 36509	41508 - 41836	54855 - 55088	62178 - 62456	
T. rubripes	*TMT-opsin1-like*	scaffold 204	250642 - 251014	253967 - 254295	260955 - 261233	257923 - 258156	
T. rubripes	*TMT-opsin2-like*	scaffold 42	784922 - 785276	780046 - 780371	775692 - 775925	771258 - 771482	
T. rubripes	*TMT-opsin3-like*	scaffold 132	153216 - 153483	154091 - 154419	155382 -155615	155729 - 155755	
T. rubripes	*TMT-opsin3-like*	scaffold 71	949293 - 949575	948958 - 949126	948697 - 948868	948391 - 948615	948251 - 948277
T. rubripes	*Opn3-like*	scaffold 52	1422232 - 1422478	1420409 - 1420728	1417768 - 1418019	1416836 - 1417057	
O. anatinu	*TMT-opsin2-like*	contig17273	5894 - 6217	4228 - 4338			
M. eugenii	*TMT-opsin2-like*	scaffold130694	93 - 123	5519 - 5844			
